# Response of Human Liver Tissue to Innate Immune Stimuli

**DOI:** 10.3389/fimmu.2022.811551

**Published:** 2022-03-09

**Authors:** Xia Wu, Jessica B. Roberto, Allison Knupp, Alexander L. Greninger, Camtu D. Truong, Nicole Hollingshead, Heidi L. Kenerson, Marianne Tuefferd, Antony Chen, David M. Koelle, Helen Horton, Keith R. Jerome, Stephen J. Polyak, Raymond S. Yeung, Ian N. Crispe

**Affiliations:** ^1^ Department of Laboratory Medicine and Pathology, University of Washington, Seattle, WA, United States; ^2^ Department of Medicine, University of Washington, Seattle, WA, United States; ^3^ Vaccine and Infectious Diseases Division, Fred Hutchinson Cancer Research Institute, Seattle, WA, United States; ^4^ Department of Surgery, University of Washington, Seattle, WA, United States; ^5^ Infectious Diseases and Vaccines, Janssen Research and Development, Beerse, Belgium; ^6^ Department of Translational Research, Benaroya Research Institute, Seattle, WA, United States; ^7^ Department of Global Health, University of Washington, Seattle, WA, United States

**Keywords:** liver slice culture, innate immunity, hepatitis C virus, direct-acting antiviral treatment, inflammation, fibrosis

## Abstract

Precision-cut human liver slice cultures (PCLS) have become an important alternative immunological platform in preclinical testing. To further evaluate the capacity of PCLS, we investigated the innate immune response to TLR3 agonist (poly-I:C) and TLR4 agonist (LPS) using normal and diseased liver tissue. Pathological liver tissue was obtained from patients with active chronic HCV infection, and patients with former chronic HCV infection cured by recent Direct-Acting Antiviral (DAA) drug therapy. We found that hepatic innate immunity in response to TLR3 and TLR4 agonists was not suppressed but enhanced in the HCV-infected tissue, compared with the healthy controls. Furthermore, despite recent HCV elimination, DAA-cured liver tissue manifested ongoing abnormalities in liver immunity: sustained abnormal immune gene expression in DAA-cured samples was identified in direct *ex vivo* measurements and in TLR3 and TLR4 stimulation assays. Genes that were up-regulated in chronic HCV-infected liver tissue were mostly characteristic of the non-parenchymal cell compartment. These results demonstrated the utility of PCLS in studying both liver pathology and innate immunity.

## Introduction

We recently reported the development of PCLS as an immunological platform (1, 2). Compared with other approaches, the PCLS method has the advantage that hepatocytes and the major subsets of non-parenchymal cells (KC, LSEC and HSC) are cultured together in their normal anatomical relationships, enabling the analysis of liver cell function in the context of intercellular interaction. Furthermore, the patterns of liver disease that are present in fresh tissue are maintained over time in the slice cultures (1). For example, steatotic liver slices retained fat droplets, and cholestatic liver slices displayed yellow-green pigment in hepatocytes. However, it remains unclear whether PCLS has the capacity to reveal alternations in innate immunity that may occur in pathological liver tissue as compared with healthy controls. As a proof of principle, we used PCLS to compare the dynamic responses of liver tissue collected from three groups of patients which included non-infected subjects, chronic HCV-infected patients, and patients whose HCV infection was cured by DAA treatment.

HCV-infected liver tissue is of particular interest, because HCV infection is one of the most common liver diseases in the USA and globally. Approximately 71 million individuals are infected with HCV worldwide (3). The acute phase of infection is often subclinical, but 55–85% of infected individuals develop a chronic infection leading to progressive liver pathology (4). The World Health Organization (WHO) estimated that approximately 399,000 people died from HCV- related diseases in 2016, mostly from cirrhosis and hepatocellular carcinoma (HCC).

The recent introduction of highly effective direct-acting antiviral (DAA) drugs has presented a unique opportunity to investigate if the diseased liver returns to normal after HCV elimination. The extent to which such drugs revert the liver to normal is not fully resolved, with some studies suggesting that HCV clearance with DAA therapy leaves residual abnormalities in innate immunity in peripheral monocytes and NK cells (5, 6), adaptive immunity in peripheral CD4+ and CD8+ T cells (7–10), in γδ-T cells (11), in mucosal associated invariant T cells (12), and in the persisting risk to hepatocellular cancer (13–16). Other studies also found abnormal serum lipids (17), persistent epigenetic modifications (18–20), and sustained hepatic inflammation (21) in liver biopsies after DAA treatment. To our knowledge, no studies have evaluated hepatic innate immunity of untreated versus DAA-treated HCV infection in the context of intact liver tissue.

## Materials and Methods

### Liver Samples, Preparation, and Culturing

Fresh non-tumor liver tissues were obtained from patients undergoing liver resection at the University of Washington Medical Center (Seattle, WA, USA). All patients in this study prospectively consented to donate liver tissue for research under the Institutional Review Board protocol #00001852. Tissue from DAA-treated patients were collected from those who achieved sustained virologic response (SVR), with HCV viral load being non-detected after the completion of DAA therapy. 7 HCV-infected, 10 DAA-cured, and 11 non-infected controls were studied. The non-infected controls are subjects with no known history of HCV exposure, their HCV negative status verified by testing their liver tissue for HCV RNA. Clinical details of these patients are provided in **Table S1**.

PCLS was performed as previously described (1). Briefly, liver cores of 6 mm diameter were excised from the resected liver tissue using a biopsy punch (Integra Miltex, York, PA, USA), stored in BELZER-UW solution (Bridge to Life Ltd., Columbia, SC, USA), and transferred to the research laboratories typically within 1 h of tissue excision. Slices 250 μm thick were prepared using a vibrating microtome, Leica VT1200 S (Nussloch, Germany), using Dulbecco’s Modified Eagles Medium (DMEM) as the cutting medium. Liver slices were cultured individually on 0.4 µm millicell organotypic inserts in 24 well plates (Millipore Corporation, Billerica, MA, USA). The culturing medium comprised 1 × advanced-DMEM medium, 5% Fetal Bovine Serum (FBS), 1 × GlutaMAX, 0.5 × Penicillin-Streptomycin, 1 × Insulin-Transferrin-Selenium supplement and 15 mM HEPES (pH 7.2 - 7.5) (all from Gibco, Grand Island, NY, USA). The liver cultures were maintained on a rocking platform at 17 rpm in a humidified incubator at 5% CO_2_ and atmospheric concentration of O_2_ at 37°C. The medium was renewed every two to three days.

### Histology of Liver Slices

Liver slices were fixed with 10% neutral-buffered formalin at room temperature for 24 h. The fixed liver slices were embedded in paraffin and were sliced into 4 μm-thick sections. Trichrome stain, picrosirius red stain, and hematoxylin and eosin (H&E) stain were analyzed with the standard protocol in the Pathology Research Services Laboratory at Department of Laboratory Medicine and Pathology at UW. The fibrosis score of liver slices was analyzed by a pathologist blinded from the patient clinical diseases. The Scheuer/Batts-Ludwig method was used for fibrosis scoring, with 0: No fibrosis, 1: portal fibrosis, 2: peri-portal fibrosis, 3: bridging fibrosis, and 4: cirrhosis. The whole-slide images were recorded with Nanozoomer Whole Slide Scanner (Hamamatsu City, Shizuoka Pref., Japan) and visualized with NDP.view2 software (Hamamatsu).

### Liver Perfusion and Isolation of Liver Cells

Human liver cell isolation procedures were adapted from several sources (22, 23). Cell isolation was performed on perfused tissue wedges, rather than cores which do not maintain adequate vasculature to allow perfusion. Perfusion buffer contained 1 × Hank’s Balanced Salt Solution (HBSS, without Ca++, Mg++, or phenol red, from Gibco), 10 mM HEPES (pH 7.2-7.5) and 0.5 mM EDTA (pH 8.0). Collagenase buffer contained 1 × HBSS (Gibco), 5 mM MgCl_2_, 5 mM CaCl_2_, 5 mM HEPES (pH 7.2-7.5), 0.5% w/v Collagenase IV (Sigma-Aldrich, St. Louis, MO, USA), 0.25% w/v Protease (Sigma), 0.125% w/v Hyaluronidase (Sigma), 0.05% w/v DNase I (Sigma). Fresh aliquots of enzymes were added to the buffer on the day of the perfusion experiment. Forty mL of Perfusion buffer and 20 mL of Collagenase buffer were used for each 10 g of liver tissue. Buffers were pre-warmed to 37°C prior to the perfusion step.

Liver tissue wedges were sequentially perfused with Washing buffer (1 × HBSS and 10 mM HEPES, pH 7.2-7.5), Perfusion buffer, Washing buffer, and the recirculating Collagenase buffer at a flow rate of 12 mL/min (Gilson’s MINIPULS 3, Middleton, WI, USA). The perfused liver tissue samples were gently mashed with a sterile syringe plunger through a sterile mesh strainer in ice-cold DMEM medium. Cell extracts were filtered through a 100 μm sterile strainer and centrifuged at 50 × *g* at 4°C for 3 min to enrich for hepatocytes in the pellets and non-parenchymal cells in the supernatant. The supernatants were transferred to a new tube and kept on ice. The pellets were washed three times with ice-cold DMEM medium, and were pelleted each time at 50 × *g* at 4°C for 3 min. To further improve the purity of the isolated hepatocytes, cell pellets were resuspended with 5 mL of ice-cold PBS, overlaid with 10 mL of 25% Percoll gradient solution. The mixture was centrifuged at 1400 × *g* (no brake) at 4°C for 20 min. The pellet contained the purified live hepatocytes. The viability of the isolated hepatocytes was determined with the trypan blue exclusion assay (Thermo Fisher Scientific, Waltham, MA, USA). If the viability was greater than 50%, the isolated hepatocytes were stored for RNA extraction.

For the non-parenchymal cells (NPC), the 50 x *g* centrifuge supernatants were further centrifuged at 500 × *g* at 4°C for 7 min. The pellets were resuspended in 5 mL of ice-cold PBS, and overlaid on top with 50% and 25% Percoll gradients (10 mL layers each), and were centrifuged at 1400 × *g* (no brake) at 4°C for 20 min. Cell layers were collected into 20 mL PBS each, and centrifuged again at 500 × *g* at 4°C for 7 min. A fraction of the pellets were saved for RNA extraction, which were the total NPC. The rest of the pellets were resuspended with the ice-cold Flow buffer containing 1 × PBS, 2% FBS, and 1 mM EDTA. The experimental protocol is illustrated in **Figure S1**.

### Flow Cytometry and Cell Sorting

The isolated non-parenchymal cells were stained with an antibody mixture that included eBioscience (San Diego, CA, USA): anti-CD45 (Cat. No. 15-0459-42); BioLegend (San Diego, CA, USA): anti-CD3 (Cat. No. 317330), anti-CD11b (Cat. No. 553310), anti-CD14 (Cat. No. 301834), anti-CD31 (Cat. No. 303120), anti-CD32 (Cat. No. 303206), anti-CD68 (Cat. No. 333814), and anti-CD271 (Cat. No. 345110). In addition, cells were also stained with LIVE/DEAD Fixable Far Red Dead Cell Stain Kit (Cat. No. L10120, Life Technologies, Carlsbad, CA, USA). The incubation mixture was kept at 4°C for 30 min in the dark on a rocking platform. The mixture was centrifuged with 500 × *g* at 4°C for 7 min. Cells were washed once with Flow buffer.

The antibody-labeled cells were sorted with a BD Aria III (BD Biosciences, San Jose, CA, USA). Analysis of cell populations was performed using FlowJo software, version 9.8.5 (FlowJo, LLC, Ashland, OR, USA). Kupffer cells were selected as the CD45+, CD3−, CD14+, CD68+, CD32+ populations (24, 25). LSECs were selected as CD45−, CD31+, CD11b− (26). HSCs were selected as CD45−, autofluorescence positive with the emission wavelength at 460 nm, SSC-H (high) (27, 28) (**Figure S1**). Cells were pelleted at 500 × *g* at 4°C for 7 min, and were stored in -80°C before RNA extraction.

### RNA Isolation and qRT-PCR Analysis

The RNA of liver slices or the purified liver cells was isolated with TRIzol and the Direct-zol RNA MiniPrep Kit (Zymo Research, Irvine, CA, USA). The RNA concentration was measured with nanoDrop (Thermo Fisher Scientific). The cDNA was synthesized with the QuantiTect Reverse Transcription Kit (Qiagen, Hilden, Germany).

A pre-amplification was performed before the multiplex qRT-PCR assays to improve the detection sensitivity (29, 30), which included PCR reactions of cDNA as templates, and the 0.2 fold- diluted primer mixture of TaqMan assays of interest (Thermo Fisher Scientific) and the BIO-X-ACT Short Mix reagents (Bioline USA Inc., Taunton, MA, USA). The pre-amplified samples were diluted five-fold with RNase- and DNase-free H_2_O. Samples were analyzed with a 48 × 48 dynamic array and a BioMark HD microfluidics system (Fluidigm, San Francisco, CA, USA). The Fluidigm Real-Time PCR Analysis software was used to calculate Ct thresholds, using the settings of quality threshold 0.65, baseline correction linear, Ct threshold method auto detection. Gene abundance of individual liver slices was normalized to the arithmetic mean of Ct values of ACTB, HPRT, and GAPDH. Typically, three liver slices were analyzed for each subject for each time point. The arithmetic mean delta Ct of three liver slices was used in the figures and for statistical analysis. The hierarchical clustering was analyzed with Cluster 3.0 (31). Heat map was visualized with Java Treeview (version 1.1.6r4) (32).

### HCV RNA Measurement

HCV RNA was measured with a Food and Drug Administration-approved Abbott Real-Time HCV assay (Abbott Molecular, Des Plaines, IL, USA) as previously described (21, 33). Fifty ng of total RNA for each sample was input in the assay, and HCV IU/ng total RNA was calculated from the calibration curve of positive controls. The reliable detection limit for HCV with this assay was 1.2 IU/50 ng liver total RNA.

### Validation of Liver Cell Types With Cell-Type-Specific Genes

Liver cell-type-specific genes for hepatocytes, Kupffer cells (KC), liver sinusoidal endothelial cells (LSEC) and hepatic stellate cells (HSC) were obtained from previous microarray analysis of liver FACS purified cells (1) and recent publications of liver single cell RNAseq (34, 35). Enrichment of the FACS purified liver cell types were confirmed by the selective expression of cell-type-specific genes.

### *Ex Vivo* Stimulation of Liver Slices With Poly-I:C and LPS

Liver slices were cultured *ex vivo* for 7 days. Final concentrations of 15 μg/mL polyinosinic–polycytidylic acid (poly-I:C) (Sigma, Cat. No. P1530), 1.5 μg/mL lipopolysaccharide endotoxin (LPS) (Sigma, Cat. No. L2630) or 1 × PBS (control, matched by volume) were added to the growth medium. During the stimulation, liver slices were maintained on transwell inserts placed on a rocking platform at 37°C. Three liver slices were harvested at each time point, including 0 h (time zero control), 2 h, 4 h, 8 h, 12 h or 24 h. The concentrations of poly-I:C and LPS treatments were based on our previous studies with isolated liver leukocytes (36) and liver slices (1).

### Archival Data

**Figure 1:**
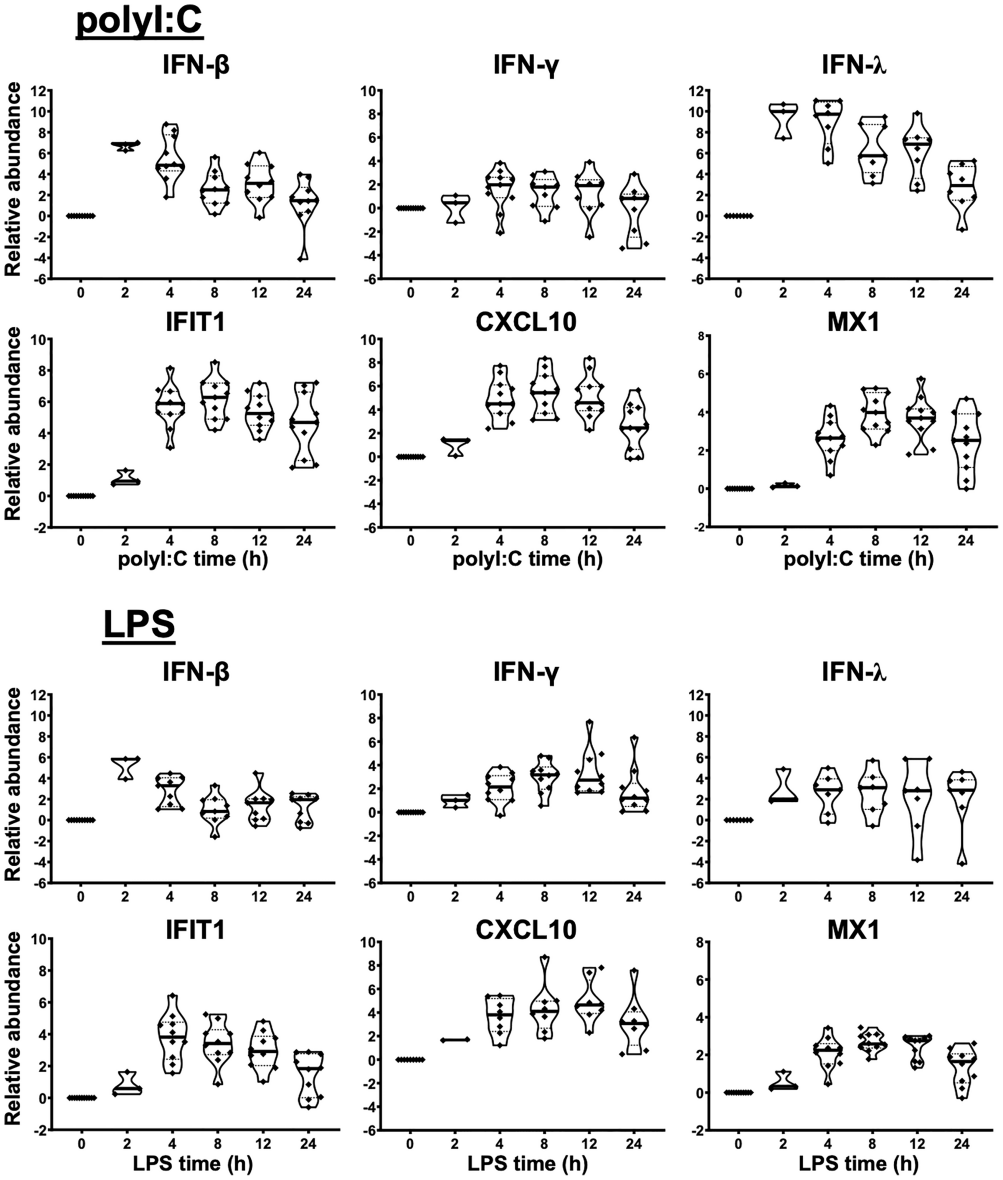
Innate immune response of human liver slices to poly-I:C or LPS treatment. Non-HCV-infected specimens are shown. IFN-β and IFN-λ expression peaked at 2-4 h, whereas interferon-stimulated gene expression for IFIT1, CXCL10 and MX1 peaked round 4-12 h. Each marker represents a patient time-point summary which consist of 2-3 biological replicates of liver slices. Relative abundance at the log2 scale is shown, with the median, 25% and 75% quantile values indicated with violin plots.

Some gene expression data from a subset of the non-infected control subjects (#1,3,4,5,6) in **Figures 1**, **5** and **S2**, **S7** were previously published in a proof-of-concept study (1), and appear here in the pool of control data. Inclusion of these data expands the number of non-infected controls, allowing us better to resolve differences due to active or DAA-cured HCV infection. For genes that were evaluated in both data-sets, we include direct comparisons in **Figure S9**. The archived data were fully consistent with newer data, justifying their inclusion.

### Statistical Significance Test

The non-parametric Mann Whitney test, Kruskal-Wallis test with Dunn’s post test with multiple test comparison correction, and Wilcoxon matched-pairs signed rank test were performed using Prism (version 9.1.0) (GraphPad Software Inc., CA, USA). Multiple comparison correction was not applied in the gene expression analysis, because a specific set of genes were targeted for analysis unlike the standard procedure in a data-driven functional genomics study. In this circumstance, correction for type I error is not advised, because the correction could lead to inflation of type II error (i.e. false negatives) (37). The issue of type II errors also arises because of the limited number of tissue samples available to us. For these reasons, we treated each gene we selected as an individual measurement.

## Results

As a first step, we verified the robust response of liver slices during poly-I:C and LPS exposure (**Figures 1**, **S2**). The 2 h time point confirmed that *IFN-β* and *IFN-λ* gene expression was stimulated prior to the elevation of interferon-stimulated genes *IFIT1/2*, *CXCL10*, *MX1* and *RSAD2* by poly-I:C. The induction of these antiviral genes was greater with poly-I:C compared with LPS treatment (**Figures 1**, **S2**). In contrast, *IL-1B* and *IL-6* and *IL-12B* were more robustly stimulated by LPS. The response of *IL-1B* and *IL-6* extended to 24 h post stimulation; in comparison, *TNF* peaked at 2 h, and gradually declined at 4, 8, 12, 24 h post-stimulation time points (**Figures 1**, **S2**). The temporal and gene specificity in poly-I:C and LPS response provided the basis for further dynamic testing of HCV-infected and DAA-cured liver tissue.

### Detection and Quantitation of HCV RNA With Liver Slices

Liver tissue from eight chronic HCV-infected patients were studied. All eight patients were diagnosed with primary liver cancer, either hepatocellular carcinoma (HCC) or intrahepatic cholangiocarcinoma (ICC). Four HCV genotypes were identified including HCV G1a, G1b, G2, G6. Likewise, liver tissue from ten DAA-cured patients were studied. The DAA-cured patients completed 8-24 weeks of DAA therapy. All subjects achieve sustained virologic response (SVR) with HCV RNA below the limit of detection after completion of DAA treatment. However, these DAA-cured patients were diagnosed with primary liver cancer (HCC or ICC) post DAA therapy. The time from completion of DAA treatment to liver resection surgery (i.e. the time liver tissue was collected) ranged from less than 1 month to 20 months, with a median of 9 month post-DAA therapy completion (**Table S1**).

**Figure 2:**
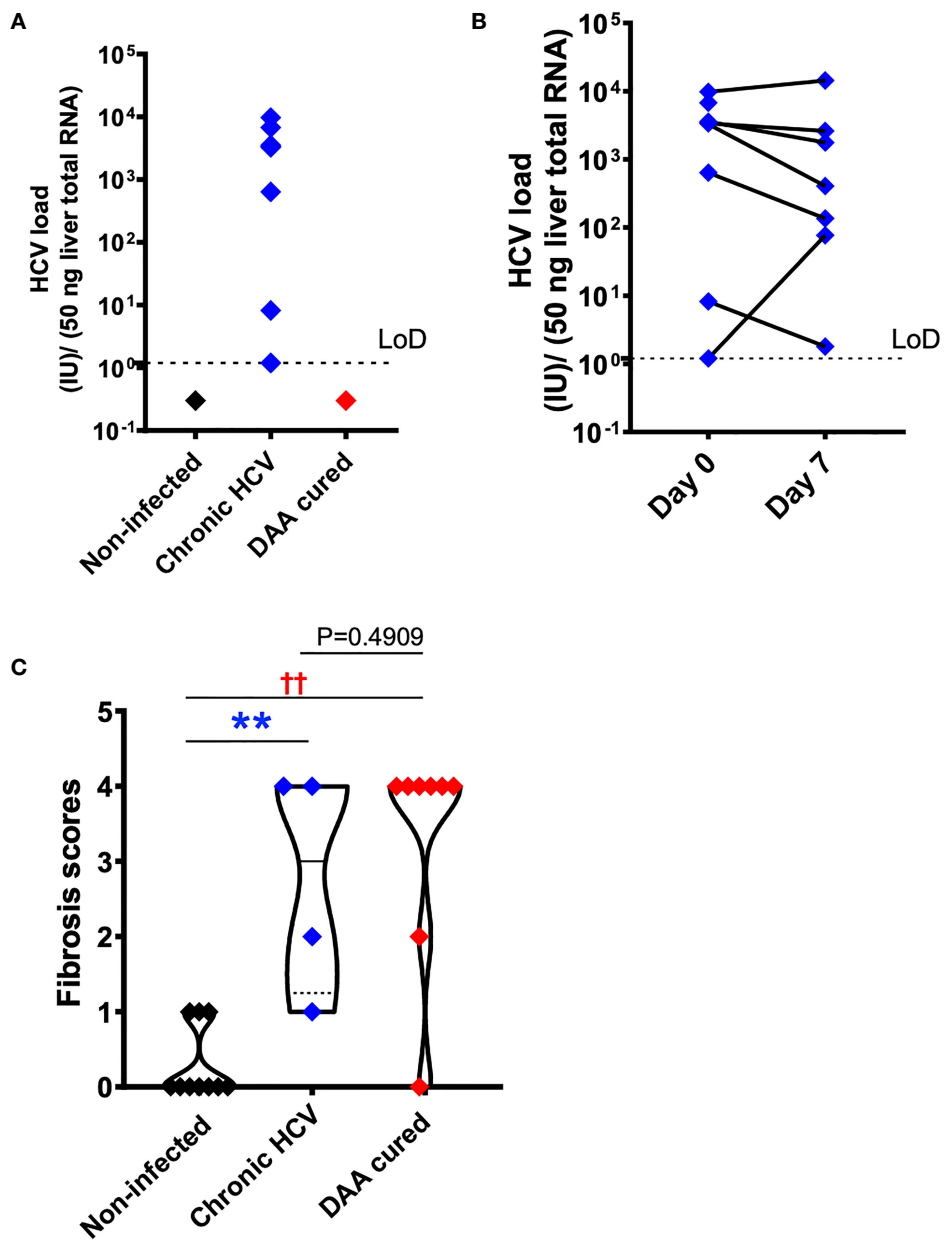
HCV and fibrosis analysis with human liver slices. **(A)** All eight chronic HCV samples (blue color) were confirmed with positive HCV RNA detection. None of the DAA cured subjects (red color) or the non-infected controls (black color) was positive for HCV RNA. The limit of reliable detection (LoD) was 1.2 IU/50 ng liver total RNA. **(B)** HCV RNA remained robustly detected in the day 7 liver slices cultured from chronically HCV-infected patients. The viral load between day 0 and day 7 liver slices was not statistically significant (P = 0.4688, Wilcoxon matched-pairs signed rank test). **(C)** Fibrosis analysis of the day 0 *ex vivo* liver specimens with trichrome staining and picrosirius red staining indicated that in DAA-treated, and now HCV-negative patients in this study there was persistent fibrosis, similar to untreated HCV and different from tissue without a history of HCV infection. The scoring system was based on the Scheuer/Batts-Ludwig method. The fibrosis score included 10 non-infected patients, 4 chronic HCV patients, and 8 DAA-treated patients. In other words, 4 out of the 7 HCV+ subjects, and 8 out of the 10 DAA-cured subjects were analyzed. Due to tissue availability, not all subjects could be analyzed in this way. The minimum and maximum data points for each subgroup are shown. Statistical significance was based on Mann-Whitney test. **P < 0.01. ††P < 0.01.

To confirm HCV infection in the samples from subjects with chronic HCV infection, HCV copy number and genotype were analyzed by UW Virology using CLIA-approved HCV assays. Liver slices originated from the chronic HCV patients were all confirmed with detectable HCV RNA, ranged from 1.2 to 9760.1 with a median value of 3325.4 IU/50 ng total RNA. In comparison, none of the non-infected or DAA-treated subjects were positive for HCV RNA *ex vivo* based on the limit of detection of 1.2 IU/50 ng total RNA (**Figure 2A**). Moreover, chronic HCV-infected liver slices continued to be HCV positive after 7 days of *ex vivo* culture (**Figure 2B** **),** with a range of 1.8-14365.8, and median of 405 IU/50 ng total RNA. The viral load on day 7 did not significantly differ from day 0 (Wilcoxon matched-pairs signed rank test, two-tailed, P=0.4688).

### Fibrosis in Chronic HCV-Infected and DAA-Cured Liver Tissue

**Figure 3:**
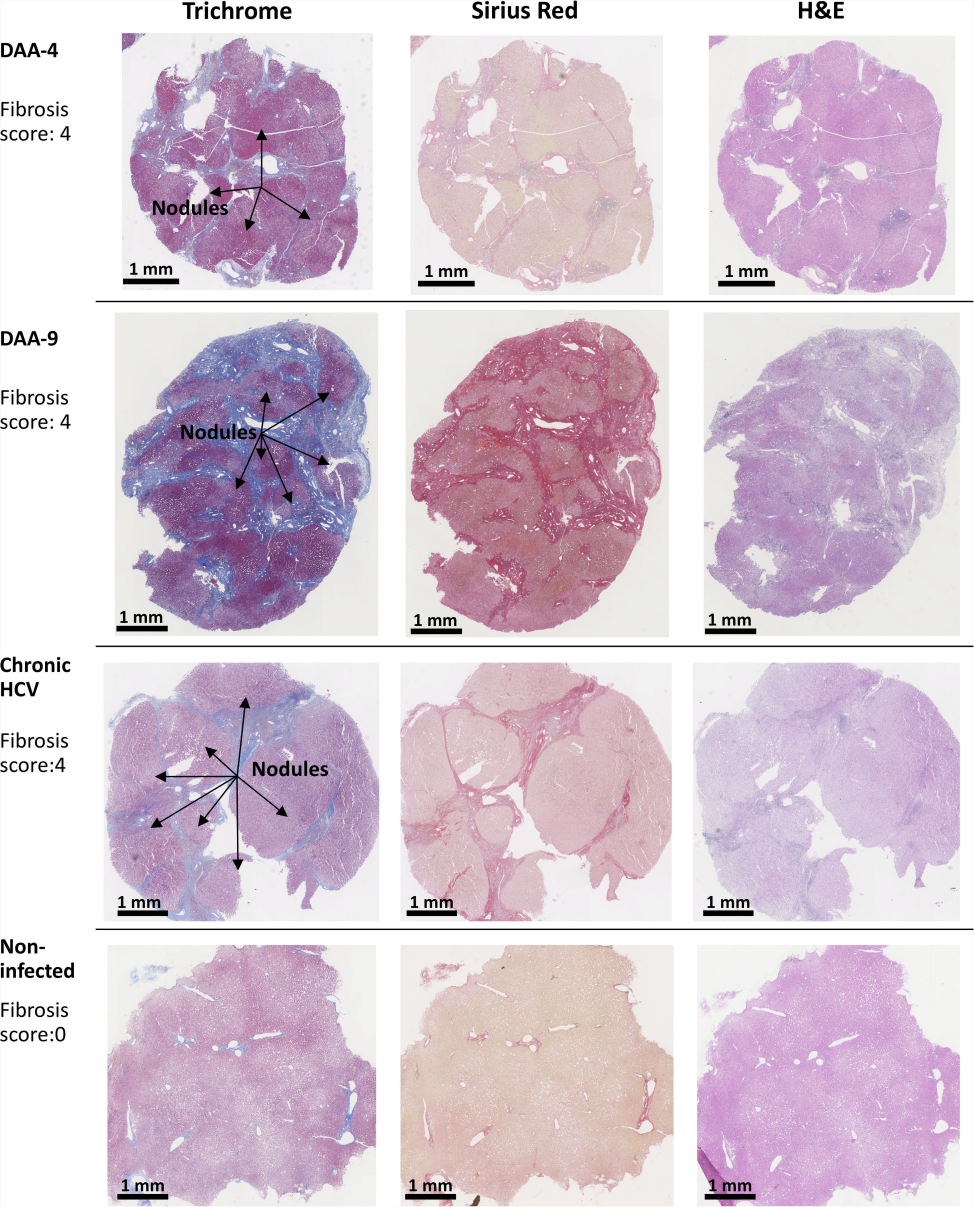
Examples of trichrome stain, picrosirius red stain, and H&E stain analysis of human liver slices. Arrow indicates the nodule formation in the cirrhosis livers. The Scheuer/Batts-Ludwig method was used for fibrosis scoring, with 0, No fibrosis; 1, portal fibrosis; 2, peri-portal fibrosis; 3, bridging fibrosis; and 4, cirrhosis. The DAA-4 liver tissue was collected 12 months after the completion of 24-week DAA therapy. DAA-9 liver tissue was collected 20 months after the completion of 12-week DAA therapy.

Because chronic HCV infection induced liver cirrhosis, we examined the liver tissue for fibrosis using picrosirius red and Masson’s trichrome staining (**Figure 2C**). Previous indirect measurements with transient elastography and noninvasive fibrosis indices reported regression of liver fibrosis after DAA treatment (38, 39). Nevertheless, direct analysis of the liver tissue revealed that six out of eight DAA-cured patients met the definition of cirrhosis (i.e., fibrosis score of 4, Scheuer/Batts-Ludwig method) in livers despite the absence of HCV RNA, even after 20 months after DAA therapy (**Figures 2C**, **3**). Likewise, the untreated chronic HCV livers were fibrotic as expected (40). None of the non-infected subjects had advanced liver fibrosis. Since the duration between the liver tissue collection and the time point of SVR ranged from less than 1 month to 20 months, it remains plausible that the fibrosis will be slowly resolved over a greater time span (41, 42). Since only the HCV+ and previously HCV+ liver tissue samples were fibrotic, an important caveat relating to our study is that the abnormal immune gene expression in both the HCV-infected and the DAA-cured liver slices could result from HCV infection, as a consequence of fibrosis, or from cross-talk between these two pathological processes.

### Immune Gene Abnormality in Chronic HCV-Infected and DAA-Cured *Ex Vivo*

**Figure 4:**
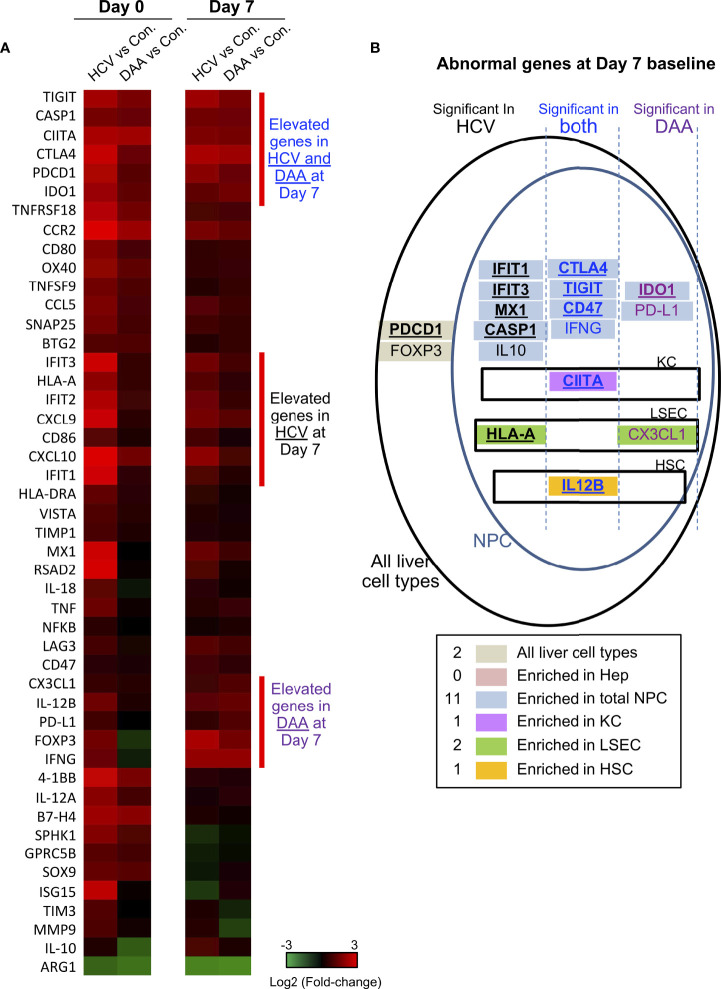
Immune gene changes at the day 7 baseline in chronic HCV-infected and DAA-cured livers. **(A)** Hierarchical clustering of immune genes with statistically significant elevation in HCV or DAA-cured subjects at Day 0 or Day 7 time points. The group-level fold changes are shown, which were calculated with the mean Ct value of each gene within each patient group. Decreased fold changes are colored in green, and increased fold changes are colored in red. **(B)** The up-regulated genes in HCV and DAA-cured at day 7 time points. Genes were mapped to liver cell types according to the cell type analysis with day 0 non-infected livers. Genes are grouped by expression patterns in cell compartments and in specimen type. The overlapped genes up-regulated at both day 0 and day 7 time points are bolded and underscored.

We used multiplex qRT-PCR to examine the expression of 140 cellular genes with functions in immune activation and suppression, and tissue repair. Forty immune genes differed in expression in chronic HCV-infected livers versus non-infected patients (Mann Whitney test, two-tailed, P < 0.05) (**Figure 4A**). This included antiviral interferon (IFN)-stimulated genes (ISGs) including *IFIT1/2/3, ISG15, RSAD2, MX1, CXCL9, CXCL10*, consistent with previous reports (43–46). In addition, immune activation genes including MHC genes *HLA-A, HLA-DRA*, *CIITA*, *CD80, CD86*, tumor necrosis factor (TNF) superfamily genes *OX40, 4-1BB, TNFSF9, TNFRSF18* and inflammation genes including *CASP1, IL-12A, IL-12B, TNF, IL-18*, and *NFKB* were also significantly up-regulated in HCV-infected livers (**Figure 4A**).

DAA treatment (i.e., HCV cure) restored 28/40 of the antiviral genes to baseline, as reported previously (47), but 11 immune genes remained elevated after cure of HCV infection. These included immune activation genes (*HLA-A, CIITA, 4-1BB, CCL5, CCR2*), inflammatory genes (*CASP1, IL-12A*), and immune suppression-associated genes (*CTLA4, TIGIT, IDO1*). Another immune suppression-related gene *ARG1* was significantly decreased in HCV+ and DAA-cured livers, compared with non-infected controls (**Figure 4A** and **Table 1**).

**Table 1:** Immune gene changes *ex vivo* (Day 0).

	Fold change			Statistical significance^2^			Cell-type-specific information^3^
	^1^HCV/ Con	DAA/ Con	DAA/HCV	HCV/ Con	DAA/ Con	DAA/HCV	
**Antiviral genes**							
Cured by DAA at D0 baseline^4^							
CXCL10	54.39	2.93	0.05	0.0003	0.4598	0.0004	NPC, KC
RSAD2	7.68	1.05	0.14	0.0007	0.7197	0.0007	NPC
IFIT1	7.16	1.51	0.21	0.0007	0.211	0.002	NPC
IFIT3	7.02	1.61	0.23	0.0002	0.0653	0.0004	NPC
CXCL9	6.80	1.45	0.21	0.0016	0.7197	0.0017	NPC, HSC
IFIT2	5.39	1.82	0.34	0.0007	0.0535	0.033	NPC, HSC
ISG15	6.16	1.04	0.17	0.0115	0.8421	0.0012	likely NPC, HSC
MX1	7.05	1.01	0.14	0.0004	0.9682	0.0002	likely NPC
**Immune activation genes**							
NOT cured by DAA at D0 baseline^5^							
HLA-A	3.84	1.61	0.42	0.0002	0.022	0.0012	NPC, LSEC
CCL5	3.30	1.92	0.58	0.0033	0.022	0.0878	NPC
CCR2	9.93	4.45	0.45	0.0007	0.0104	0.02	likely NPC
4-1BB (CD137, TNFRSF9)	6.35	3.17	0.50	0.0007	0.0279	0.0702	likely NPC
CIITA	5.03	4.64	0.92	0.0007	0.0003	0.6691	likely KC
Cured by DAA at D0 baseline							
HLA-DRA	2.53	1.43	0.56	0.0052	0.2775	0.1088	NPC
CD80	3.54	1.91	0.54	0.0012	0.1823	0.0553	NPC
CD86	2.32	1.25	0.54	0.0418	0.447	0.1613	NPC
OX40 (TNFRSF4)	3.93	2.46	0.63	0.0033	0.0789	0.3638	LSEC
TNFRSF18	5.62	2.97	0.53	0.0006	0.0745	0.351	HSC, LSEC
TNFSF9	2.97	2.08	0.70	0.036	0.2224	0.2721	likely KC
CXCL1	2.73	1.01	0.37	0.1447	0.9048	0.003	HSC
IFNG	2.69	0.83	0.31	0.1738	>0.9999	0.0431	NPC
**Inflammation genes**							
NOT cured by DAA at D0 baseline							
CASP1	3.13	2.71	0.87	0.0007	0.0435	0.0136	NPC
IL-12A	3.71	1.91	0.51	0.0037	0.0434	0.0185	LSEC
Cured by DAA at D0 baseline							
IL-12B	2.86	1.29	0.45	0.0262	0.7577	0.1738	HSC
TNF	2.77	1.06	0.38	0.0115	0.7802	0.0046	NPC, KC, HSC
IL-18	2.40	0.90	0.37	0.0164	0.6607	0.025	NPC, HSC
NFKB	1.46	1.01	0.69	0.0283	>0.9999	0.0317	N/A
**Immune Suppression genes**							
NOT cured by DAA at D0 baseline							
CTLA4	6.52	2.70	0.41	0.0003	0.0172	0.033	likely NPC
TIGIT	4.77	3.20	0.67	0.0003	0.0172	0.033	likely NPC
IDO1	4.35	2.52	0.58	0.0052	0.0435	0.1613	NPC
ARG1	0.45	0.39	0.89	0.0229	0.0056	0.6806	Hep, uncommon in KC, LSEC
Cured by DAA at D0 baseline							
B7-H4	4.49	3.70	0.83	0.0059	0.0541	0.7104	HSC
VISTA (VSIR)	2.10	1.40	0.67	0.0021	0.1333	0.3638	KC, LSEC, HSC
CD47	1.43	1.24	0.87	0.0418	0.3562	0.3148	NPC, KC, LSEC, HSC
PDCD1	5.16	2.30	0.44	0.0205	0.0592	0.1416	not cell-type-specific
LAG3	1.88	1.19	0.64	0.0164	>0.9999	0.0712	not cell-type-specific
TIM3	2.18	0.99	0.46	0.0079	0.8633	0.0164	NPC
FOXP3	2.94	0.70	0.24	0.1416	0.8633	0.0079	not cell-type-specific
**PI3K pathway genes**							
NOT cured by DAA at D0 baseline							
SPHK1	3.50	2.13	0.61	0.0052	0.04	0.2523	HSC
BTG2	2.19	2.10	0.96	0.0907	0.0188	0.8371	N/A
SOX9	2.62	2.30	0.88	0.0549	0.04	>0.9999	Not cell-type-specific
SNAP25	3.03	1.88	0.62	0.0164	0.0503	0.351	N/A
GPRC5B	2.30	1.91	0.83	0.0311	0.1615	0.351	N/A
**Tissue repair genes**							
Cured by DAA at D0 baseline							
TIMP1	1.98	1.29	0.65	0.0311	0.447	0.2698	LSEC
MMP9	2.04	1.13	0.56	0.0164	0.7802	0.1331	Not cell-type-specific
PDGFB	1.62	0.93	0.58	0.2523	0.8633	0.0164	LSEC
TGFB1	2.18	1.27	0.58	0.0712	0.447	0.0431	KC, LSEC, HSC

Liver tissue was perfused, and total livers, hepatocytes (Hep) and NPC populations (NPC) were obtained with differential centrifugation. Kupffer cells (KC), liver sinusoidal endothelial cells (LSEC), and hepatic stellate cells (HSC) enriched populations were obtained with FACS from NPC (**Figure 1B**). RNA was extracted from each cell type, followed by qRT-PCR gene expression analysis using the fluidigm platform.

^1^The comparisons were HCV/Con, chronic HCV-infected versus non-infected controls; DAA/Con, DAA-cured versus non-infected controls; DAA/HCV, DAA-cured versus chronic HCV-infected.

^2^Statistical significance was based on non-parametric two-tailed Mann-Whitney test.

^3^Cell-type-specific information was based on analysis of the non-HCV-infected samples. N/A means cell-type-specific gene information not analyzed. This was due to the limit of sample availability and the assay through-put with fluidigm assays.

^4^Cured by DAA at D0 baseline categorized the gene subsets that were normalized to the same level as non-infected controls.

^5^Not cured by DAA at D0 baseline categorized gene subsets that were not normalized to the correct levels as the non-infected controls.

The phosphoinositol-3-kinase (PI3K) pathway-linked genes are susceptible to modification by HCV infection (18, 20, 48) and liver fibrosis (49, 50). We found that *SPHK1, BTG2, SOX9, SNAP25*, and *GPRC5B* were significantly up-regulated in chronic HCV infected livers. Furthermore, *SPHK1, BTG2*, and *SOX9* remained significantly up-regulated after HCV clearance with DAA treatment (**Figure 4A**).

### Immune Gene Subsets Mapped to Hepatic Non-Parenchymal Immune Cells

To identify the cellular origin of the dysregulated immune gene subsets, we investigated gene expression with liver cells subsets purified using FACS-sorting (1). Six liver wedge samples of non-infected liver tissue were large enough in size and anatomically ideal for perfusion through the vasculature, which allowed them to be dissociated and analyzed for gene expression. We could not do this with samples from HCV-infected or DAA-cured patients due to limitations in the amount of liver tissue obtained. The purity of isolated liver cell types was confirmed based on the expression of cell marker genes (1, 34, 51), including *FCN1, NLRP3, ITGA4* for KC, *EMCN, VWF, CD34* for *LSEC, FGFR2, PDGFRB, COL1A1, LRAT* for HSC, and *FBP1, RBP4, ALB* for hepatocytes (**Figure S3**).

Many immune genes that were up-regulated in HCV-infected or DAA-cured fibrotic liver tissue were significantly enriched in the NPC compartment (**Table 1** and **Figure 4B**). For a smaller subset of immune genes, we were able to map them to specific immune cells with greatest expression: for example *CIITA* mapped to Kupffer cells (KC)*, HLA-A* and *CX3CL1* mapped to liver sinusoidal endothelial cells (LSEC), and *IL-12B* mapped to Hepatic Stellate Cells (HSC) (**Figures 4B**, **S4**). Even though the NPC fraction may not include the cell types targeted by HCV infection, previous studies demonstrate that NPC can be activated through paracrine signaling from the neighboring HCV-infected hepatocytes (44, 52, 53).

### Innate Immune Responses Were Altered in HCV Infected and DAA-Cured Liver Tissue

**Figure 5:**
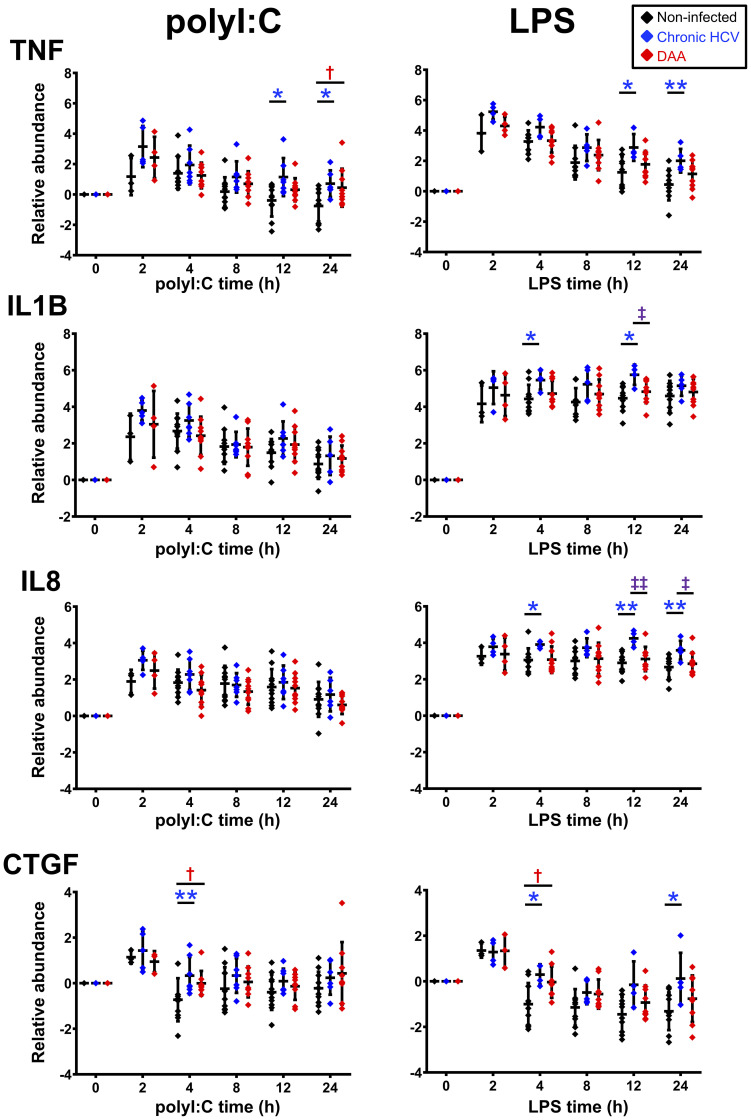
Delta-delta Ct analysis showing up-regulated genes post- poly-I:C or LPS stimulation in chronic HCV-infected and DAA-cured liver slices. The relative gene abundance was normalized to to the arithmetic mean of Ct values of ACTB, GAPDH and HPRT1. The relative abundance was further normalized to the day 7 time zero measurements. Statistical significance was based on two-tailed Mann-Whitney test. *, statistical significantly different between non-infected versus chronic HCV liver slices. †, statistical significantly different between non-infected versus DAA-cured liver slices. ‡, statistical significantly different between chronic HCV versus DAA-cured liver slices. Levels of statistical significance are indicated thus: *P < 0.05 **P < 0.01. The mean and standard deviation within each group are plotted.

We stimulated liver slices with poly-I:C and LPS for varying times using the method established in our previous study (1). We verified the immune genes activated by tissue slicing had broadly stabilized after 4 days of *ex vivo* culture for HCV-infected and DAA-cured slices (**Figures S5A, B**), similar to the non-infected slices described previously (1). Day 7 was thus an ideal time-point to add exogenous stimuli, and the expression of immune genes was recorded at the day 7 baseline (T_0_) prior to the treatment. Twelve of the immune genes remained at elevated baseline in HCV-infected or DAA-cured samples compared with non-infected control (**Figures 4A, B** **)**. The PBS mock stimulation did not significantly induce IFNs and ISGs in liver slices (1). Notably, expression of TLR3, TLR4, and NF-κB were not significantly different at day 7 among chronic HCV-infected, DAA-treated and non-infected liver slices (**Figure S6A**). We used the ΔCt method with internal normalization to *ACTB, GAPDH* and *HPRT* to evaluate the gene expression level post stimulation (**Figure S7**). We also used the ΔΔCt method against the day 7 T_0_ to assess the enhanced net increase of genes during TLR3/TLR4 response (**Figure 5**). We confirmed that TLR3 and TLR4 are primarily expressed in the NPC compartment (**Figure S6B**), consistent with the expectation that NPCs in the liver are the primary responders to these triggers (54).

These studies revealed the following findings. First, robust induction of IFNs and ISGs by both TLR3 and TLR4 agonists was observed in liver slices from all three groups of liver specimens (*i.e.*, non-infected, HCV-infected, and DAA-cured). The sequential signaling cascade induced by TLR3 and TLR4 pathways described in **Figures 1**, **S1** for non-infected liver tissue, were also pronounced in the HCV-infected and DAA-cured liver tissue. For example, *IFNB1* and *IFNL3* (IL28B) peaked at 2-4 h, prior to the maximum abundance of ISGs (*IFIT 1/2/3, RSAD2* and *MX1*) which occurred at 4-8 (**Figures 5**, **S6**).

Second, some immune genes were not suppressed but induced more strongly by TLR3 agonist in liver slices of chronic HCV-infected liver at least at one time point (Mann-Whitney test, two tailed, P < 0.05). These included antiviral genes*, MX1, RSAD2, IFNG*, chemokine genes *CCL7, CX3CL1*, and inflammatory genes *TNF* and *CASP1* (**Figure S8**). Other common activation markers for TLR3 signaling, including *IFNB1, IL28B, IFIT1/2/3, ISG15, CXCL10, IL-1B* and *CCL-5*, were also responsive in chronic HCV-infected slices with elevation of greater than 10-fold, as strong as changes in the non-infected liver slices. Thus, TLR3 sensing and signaling did not appear to be impaired in chronic HCV-infected liver slices.

**Figure 6:**
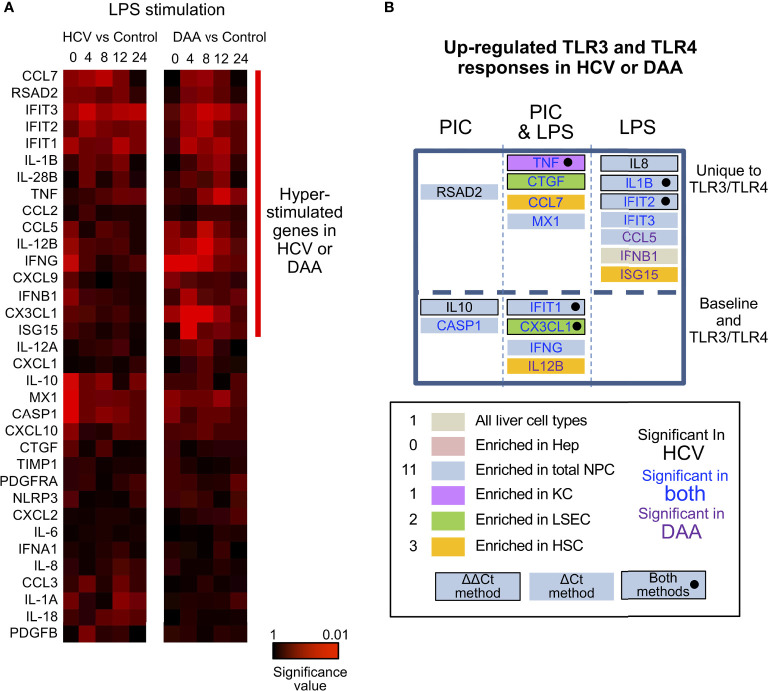
Altered TLR3 and TLR4 response in chronic HCV-infected liver slices not restored in DAA-cured liver slices. **(A)** Hierarchical clustering of immune responsive genes with 2-fold or greater induction during LPS stimulation. The statistical significance P value with ΔCt method is shown. Gene abundance of individual liver slices was normalized to the arithmetic mean of Ct values of ACTB, GAPDH and HPRT. Two-tailed Mann-Whitney test was used. Greater significance P value is colored with darker red. **(B)** Genes up-regulated with P < 0.05 at one time point in either poly-I:C or LPS treatment were included as up-regulated genes. Genes boxes were colored with the enriched expression in cell compartment of day 0 information. Using the ΔΔCt method, genes that increased in expression during TLR3/TLR4 response are highlighted with a black bordering; genes shown to increase based on both ΔΔCt and ΔCt methods are highlighted with black bordering and black solid circles. Genes names are colored by in HCV- or DAA- samples or both. These genes were further grouped by whether they were differentially expressed in TLR3 or TLR4 treatment not at the baseline level, and whether poly-I: C or LPS treatment revealed differential responses.

TLR4 sensing and signaling were similarly robust in chronic HCV-infected livers. *IFIT1/2/3* and *CCL7* were identified at greater abundance post LPS stimulation in chronic HCV-infected liver slices on at least one time point (Mann-Whitney test, two tailed, P < 0.05) (**Figures 6A**, **S7**).

Third, most of the hyper-induction of the genes in HCV-infected tissue (9/11 genes in TLR3 response, 10/11 genes in TLR4 response) was not reversed in the tissue from formerly HCV+, but now successfully treated donors. *IFNB1, CCL5*, *ISG15* and *IL-12B* were more strongly induced by LPS or poly-I:C in DAA-cured tissue, previously not identified with HCV-infected tissue (**Figures 6A, B** **)**.

In terms of the ΔΔCt net increase, *TNF, IL-1B, IL-8, CTGF*, and *CX3CL1* were more strongly induced in HCV-infected liver tissue during LPS or poly-I:C stimulation. Likewise, *TNF, CTGF and IFIT2* were abnormally induced in DAA-cured liver tissue (**Figures 5**, **6B**). The abnormal induction of *TNF, IL-1B, IL-8*, and *CTGF* at 12 h and 24 h in chronic HCV liver slices, indicated persistent inflammation (**Figure 5** **)**. Importantly, a subset of these genes *TNF* in KC, *CTGF* in LSEC, *CCL7, ISG15* for HSC, and *IL-8, IL-1B, IFIT2/3, RSAD2, MX1, CCL5* for NPCs, and *IFNB1* were only revealed through the TLR3 and TLR4 stimulation assays, but not with the *ex vivo* day 0 measurements. These results highlighted the unique value to measure dynamic changes with liver tissue to reveal immune signaling defects (**Figures 6B**, **S9**).

## Discussion

In this study, we pioneered the PCLS technology to study innate immune response within normal and pathological liver tissue. We showed that immune abnormalities persist after the elimination of HCV by anti-viral therapy, and the persisting inflammatory and immune signatures featured genes characteristic of hepatic non-parenchymal cells. Innate immunity in response to TLR3 and TLR4 agonists was not suppressed but enhanced in HCV-infected tissue, and these abnormalities were not corrected after effective DAA treatment.

In human hepatocyte cultures (55, 56) HCV infection triggered cleavage of MAVS and TRIF host proteins by HCV NS3/4A protease, disrupting TLR3-antiviral signaling. Reduction of protein levels of MAVS and TRIF was also observed previously in chronic HCV-infected livers (57, 58). The novel aspect of PCLS is to study whole liver slices consisting of hepatocytes (infected cells) and non-parenchymal cells (NPC, non-infected cells). We observed a robust response to TLR3 signaling in the HCV-infected liver, which supports the idea that ISG induction may be weak in HCV infected hepatocytes, but is strong in HCV infected liver due to paracrine activation of NPC. The data on the direct effect of HCV on the TLR3 response in liver tissue are novel and distinct from previous work focused on the IFN-induced gene expression in chronically HCV-infected liver by pegylated IFN therapy (59, 60), since such direct activation of IFN signaling bypasses MAVS and TRIF.

A previous study reported that HBV does not interfere with innate immune response based on chronic HBV-infected liver biopsy tissues (61). However, in chronic HCV-infected tissue, we found innate immunity *via* TLR3 and TLR4 was not suppressed but enhanced. The different outcome may reflect the differences in immune evasion mechanisms between DNA and RNA viruses. Furthermore, the tissue status and treatment method were not identical between the two studies. In chronic HCV-infected tissue, advanced fibrosis was observed, and this study also used the day 7 as treatment time zero when immune activation by tissue slicing was broadly stabilized.

From the days of IFN-based therapy, we know that HCV-induced liver disease can regress when the virus is eradicated from the liver (62). However, halting or reversing liver disease upon IFN cure of HCV has not been universal; some patients with advanced disease still go on to develop HCC (63–65). DAA drugs are now able to cure the majority of HCV infections, even in subjects with advanced liver disease. Nevertheless, clearance of HCV with DAA treatment may only partially restore immune cell function. In this study, we have identified immune genes that were restored to normal by DAA treatment, and more importantly immune genes that were not restored *ex vivo* (*i.e.*, at day 0 time point), including a subset of immune activation genes, inflammation genes, immune suppression, and PI3K pathway genes.

In North America, the era during which some, but not all HCV patients were treated with DAA was brief and has passed. Within this narrow time frame, we worked with tissue from patients who were treated for surgical resection of a liver lesion. Accordingly, there are inherent limitations to this study. First, due to the small sample size, we did not further classify patient subgroups based on HCV genotypes. Likewise, other subject characteristics including gender, age, genetic polymorphisms (such as *IL28B*), and liver fibrosis status, all of which may affect immunity and responses to HCV infection (13, 66, 67) were not used as factors to subdivide patient groups in the downstream analysis. Nonetheless, we observed significant effects that cut across these hidden variables, with consistent identification of aberrant immune gene expression at baseline day 0 and day 7, as well as those with altered TLR3 and TLR4 responses. Second, we cannot determine whether the sustained abnormality is due to the host response to hepatic fibrosis or HCC rather than HCV-mediated. The DAA-cured subjects we studied were based only on those who developed HCC or ICC post DAA therapy, since these were the patients who underwent liver resection. Thus, our DAA-cured samples only covered a unique sub-group of DAA-cured patients. Third, only KC, LSEC, HSC enriched genes could be assigned to specific liver cell type. Many other differentially expressed genes were mapped to the NPC compartment, but the analysis of isolated cells did not give us sufficient resolution to allow us to assign any genes to other liver cell types, such as CD8+ T cells, CD4+ T cells, NK cells, dendritic cells and monocytes.

Previous analysis with DAA-cured PBMC suggest both the functional phenotypes (8, 11, 68, 69) and the frequency/composition (70, 71) of immune cells are relevant aspects not fully restored by DAA therapy. Since we did not interrogate the expression of these genes in NPCs from samples other than the non-infected controls, it is unclear whether the gene expression changes were contributed mainly by the NPC cell phenotypes or by the overall greater abundance of immune cells in HCV+ and DAA-cured livers. Hence, IHC analysis of overall abundance of NPC populations in liver slices between groups would be impactful to address this question. Future study may extend the conclusion through analysis of a more diverse group of clinical situations, including chronic HCV patients and DAAs-SVR cases with or without fibrosis or HCC, as well as non-HCV related liver tissues with fibrosis or HCC. Liver tissue with longer time span post DAA therapy (>20 months) will also help clarify the resolution of liver cirrhosis by DAA therapy.

We made use of the PCLS approach opportunistically to obtain data from the group of untreated HCV-infected subjects, a group that has now disappeared from the surgical patient pool, at least in North America. Despite the heterogeneity of both subjects and controls, this study demonstrates the power of PCLS to document functional responses of intact human liver tissue.

## Data Availability Statement

The original contributions presented in the study are included in the article/**Supplementary Material**. Further inquiries can be directed to the corresponding author.

## Ethics Statement

The studies involving human participants were reviewed and approved by Institutional Review Board, University of Washington. The patients/participants provided their written informed consent to participate in this study.

## Author Contributions

XW, JR, AC, HH, RY, and IC designed research; XW, JR, AK, AG, NH, HK, MT, and AC performed research; DK, KJ, SP, and RY contributed reagents/analytic tools; XW, AG, CT, and IC analyzed data; XW, SP, and IC wrote the manuscript. All authors reviewed the manuscript and provided suggestions.

## Funding

This work was supported by NIH NIAID R56 grant AI143683, US Department of Defense grant CA150370, Janssen Research and Development, the Seattle Foundation, and the University of Washington.

## Conflict of Interest

The authors declare that the research was conducted in the absence of any commercial or financial relationships that could be construed as a potential conflict of interest.

## Publisher’s Note

All claims expressed in this article are solely those of the authors and do not necessarily represent those of their affiliated organizations, or those of the publisher, the editors and the reviewers. Any product that may be evaluated in this article, or claim that may be made by its manufacturer, is not guaranteed or endorsed by the publisher.

## Abbreviations

PCLS, precision-cut liver slice; TLR, toll-like receptor; NPC, non-parenchymal cell; KC, Kupffer cells; LSEC, Liver sinusoidal endothelial cells; HSC, hepatic stellate cells; HCV, hepatitis C virus; DAA, direct-acting antiviral; ISGs, interferon-stimulated genes; HCC, hepatocellular carcinoma; ICC, intrahepatic cholangiocarcinoma; SVR, sustained virologic response.
