# Supplementary material for: Response of Human Liver Tissue to Innate Immune Stimuli
Source: Front Immunol. 2022 Mar 9;13:811551. doi: 10.3389/fimmu.2022.811551 (PMC8959492; doi:10.3389/fimmu.2022.811551)
Supplement: Supplementary file 1 [file DataSheet_1.pdf]

Supplemental Figures

Figure S1

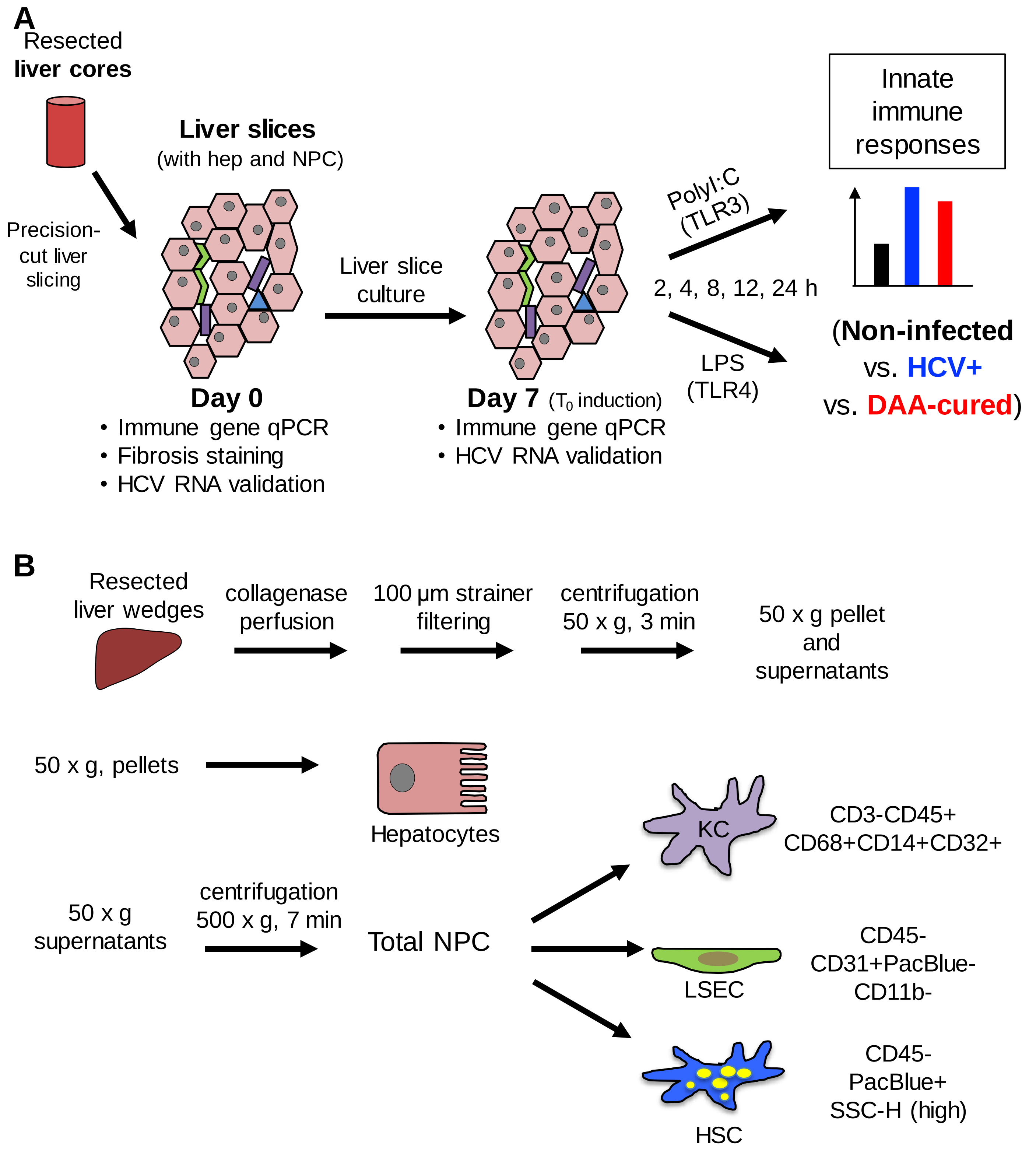

Figure S1. Experimental Overview for this study. (A) Human liver slices to study innate immune response. Liver slices were harvested on Day 0 immediately after slicing or cultured for seven days until ex vivo stimulation with TLR3 agonist poly-I:C or TLR4 ligand LPS. Innate immune responses were compared among three groups of tissue samples including non-infected patients (controls), chronic HCV-infected patients (HCV+), and patients with previous history of HCV infection who were cured by DAA treatment (DAA-cured patients). (B) Specific liver cell types were purified from fresh resected liver wedges with perfusion, differential centrifugation and fluorescence-activated cell sorting (FACS) techniques. Immune genes of interest were analyzed with liver cell samples to determine liver cell types with enriched gene expression. Hepatocytes (Hep), total non-parenchymal cells (NPC), Kupffer cells (KC), liver sinusoidal endothelial cells (LSEC), or hepatic stellate cells (HSC).

Figure S2

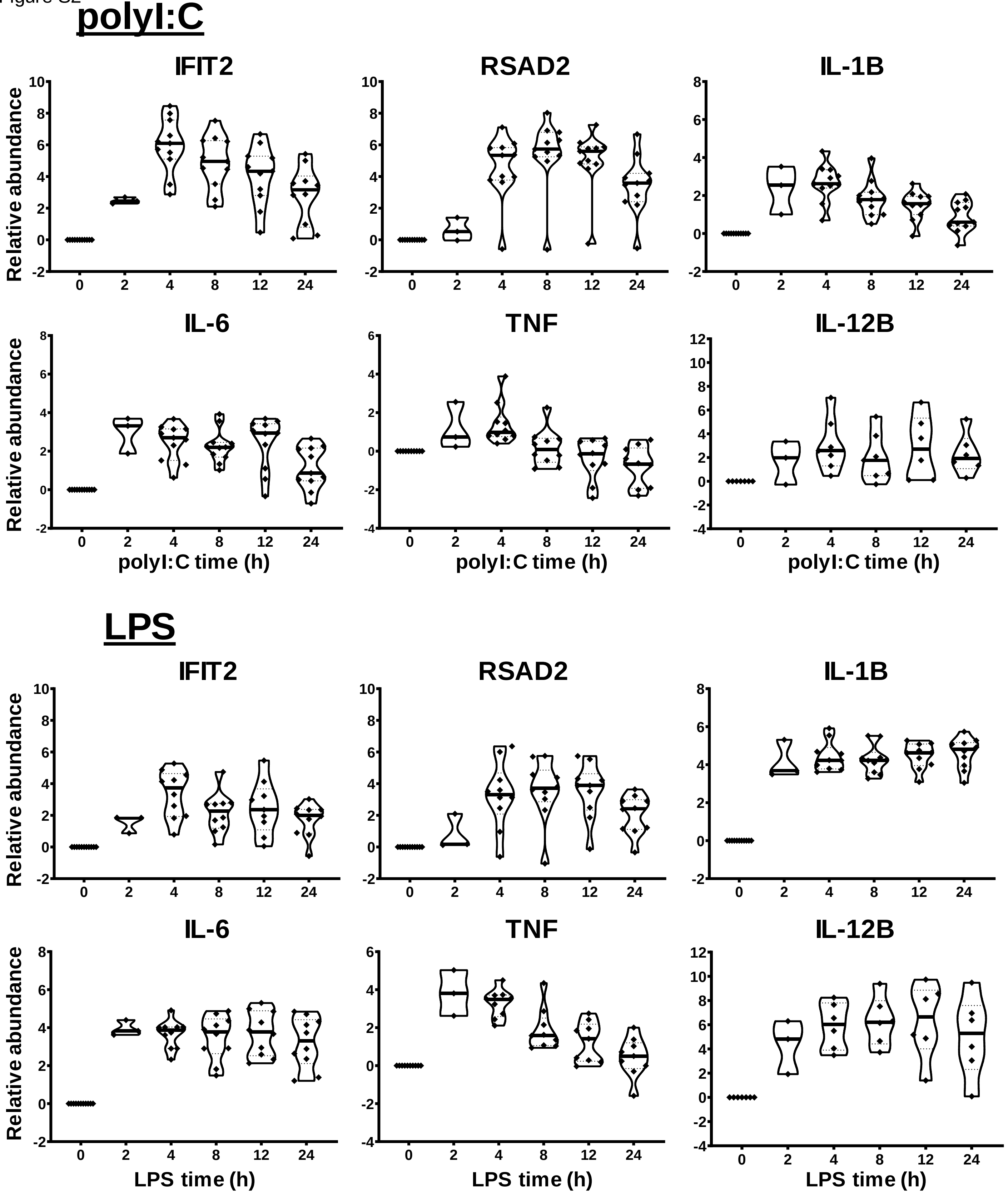

Figure S2. Additional examples for innate immune response of human liver slices to poly-I:C or LPS treatment. Non-HCV-infected specimens are shown. IL1B, IL6, TNF and IL12B expressions were more robustly stimulated with LPS treatment compared with poly-I:C, with a peak induction 2 h onwards. IFIT2 and RSAD2 peaked at 4-12 h, with greater induction by poly-I:C treatment. Each marker represents a patient time-point summary. Relative abundance at the log2 scale is shown, with the median, 25% and 75% quantile values indicated with violin plots.

Figure S3

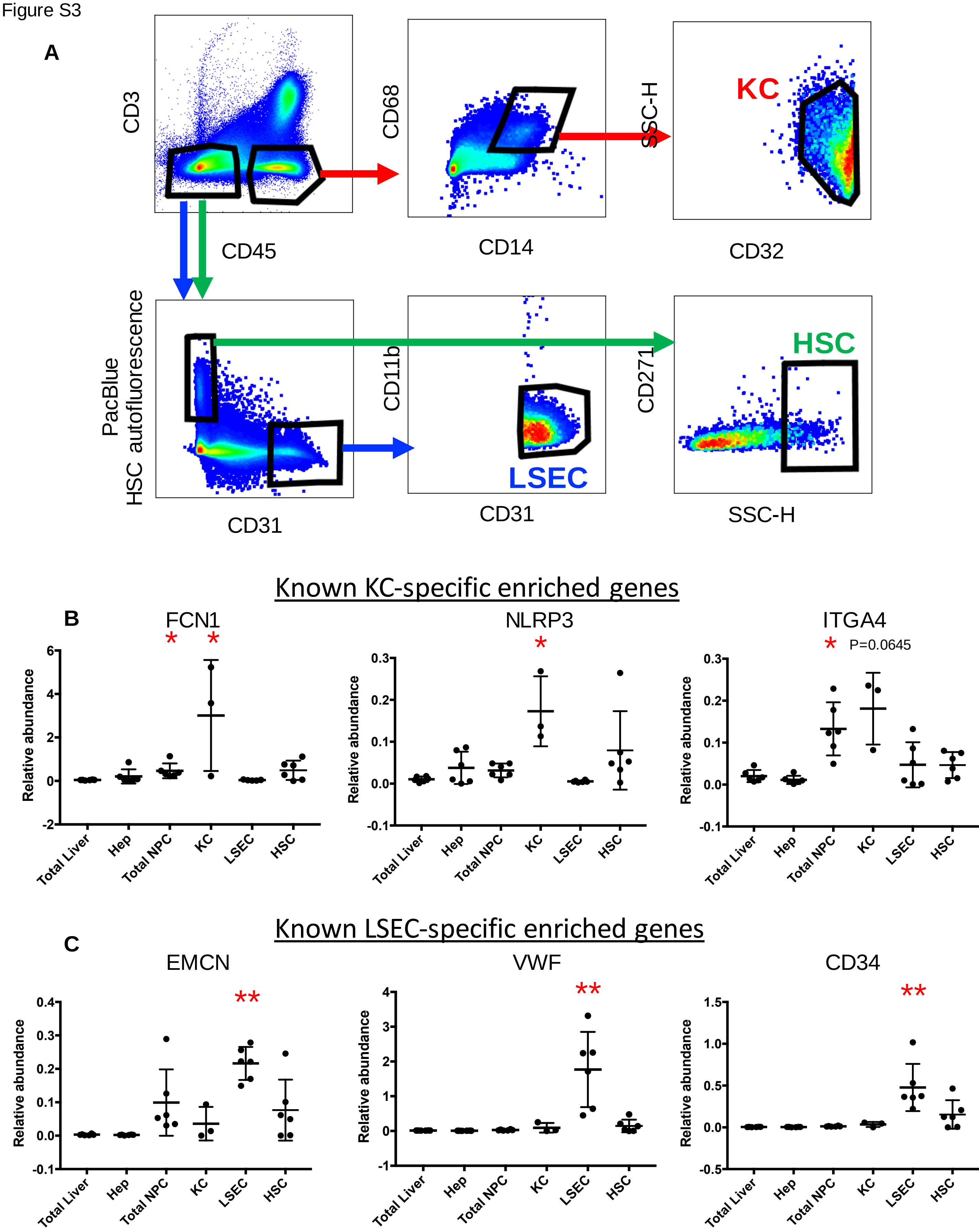
A
CD3
CD45
CD68
CD14
SSC-H
KC
CD32
PacBlue
HSC autofluorescence
CD31
CD11b
LSEC
CD31
CD271
HSC
SSC-H
Known KC-specific enriched genes
B
FCN1
NLRP3
ITGA4
*
*
*
*
P=0.0645
Relative abundance
Total Liver
Hep
Total NPC
KC
LSEC
HSC
Known LSEC-specific enriched genes
C
EMCN
VWF
CD34
**
**
**
Relative abundance
Total Liver
Hep
Total NPC
KC
LSEC
HSC

Figure S3

Known HSC-specific enriched genes

D

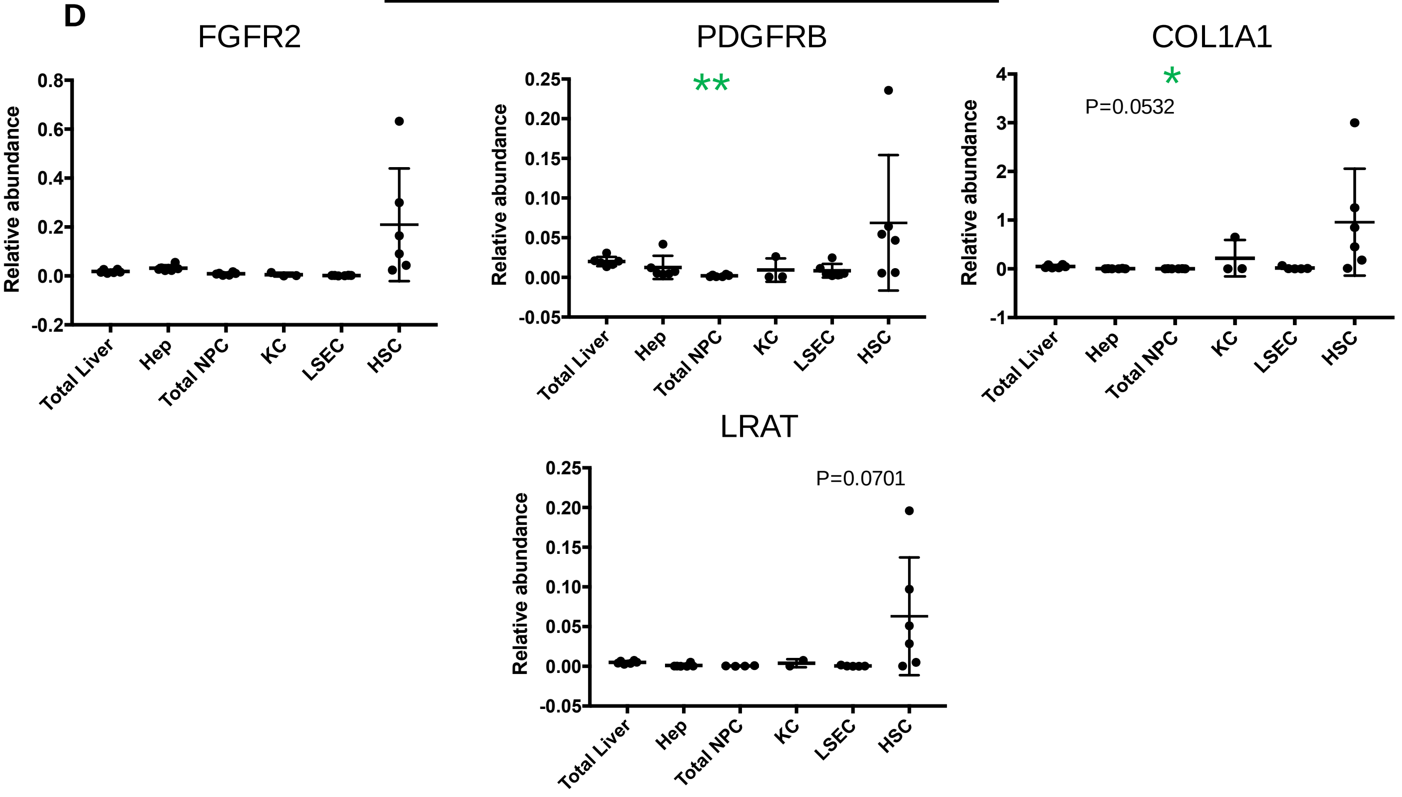

Known Hep-specific enriched genes

E

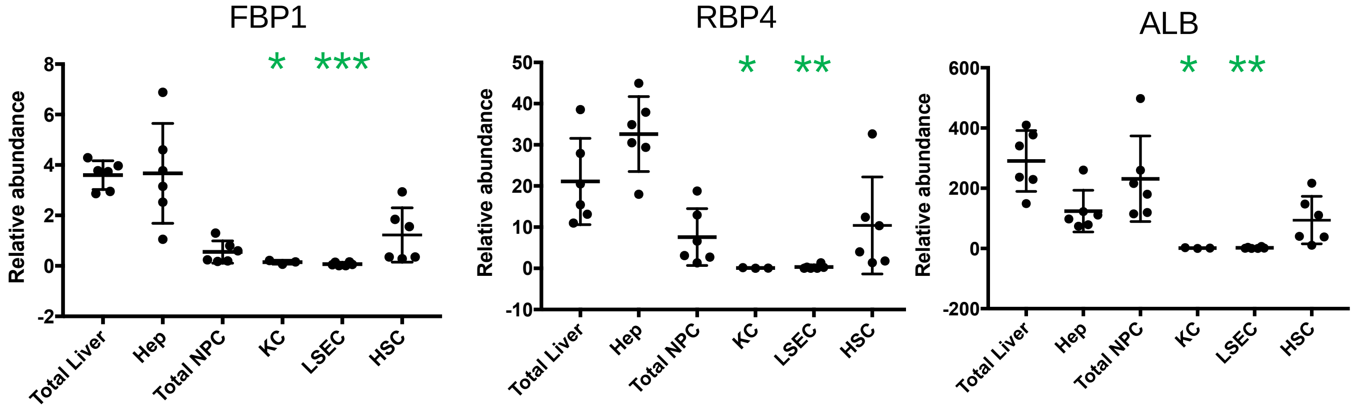

Figure S3. FACS purified liver cell types and validation. (A) Examples of FACS with non-infected liver wedge for Kupffer cells (KC), liver sinusoidal endothelial cells (LSEC) and hepatic stellate cells (HSC). Hepatocytes (Hep) and the total non-parenchymal cells (NPC) were collected after centrifugation steps prior to cell sorting. (B)-(E) Validation of purified cell populations of KC, LSEC, HSC, Hep and NPC. Cell isolation was performed on wedges of resected tissue rather than cores. Non-infected liver tissue were cell sort and analyzed for gene expression, but HCV-infected or DAA-cured patients were not analyzed due to limitations in the amount of liver tissue obtained. Each dot within each cell type represents an individual patient sample. The relative gene abundance was normalized to arithmetic mean of ACTB, GAPDH and HPRT1. The relative abundance of linear scale is shown. Statistical significance was based on Kruskal-Wallis test with Dunn's post test with multiple test comparison correction. Genes that were enriched in a cell type compared with total livers are denoted with **red** asterisks, while genes that were significantly less abundant compared with total liver are indicated with **green** asterisks. *, P <0.05 **, P<0.01, ***, P<0.001. The mean and standard deviation are plotted. Graphs and statistical test were analyzed with Prism version 9.1.0. Albumin which was thought as a good hepatocyte specific marker were detected with positive signals in cell fractions of total NPC and HSC. Thus, ALB is less hepatocyte-specific compared with FBP1 and RBP4.

Figure S4

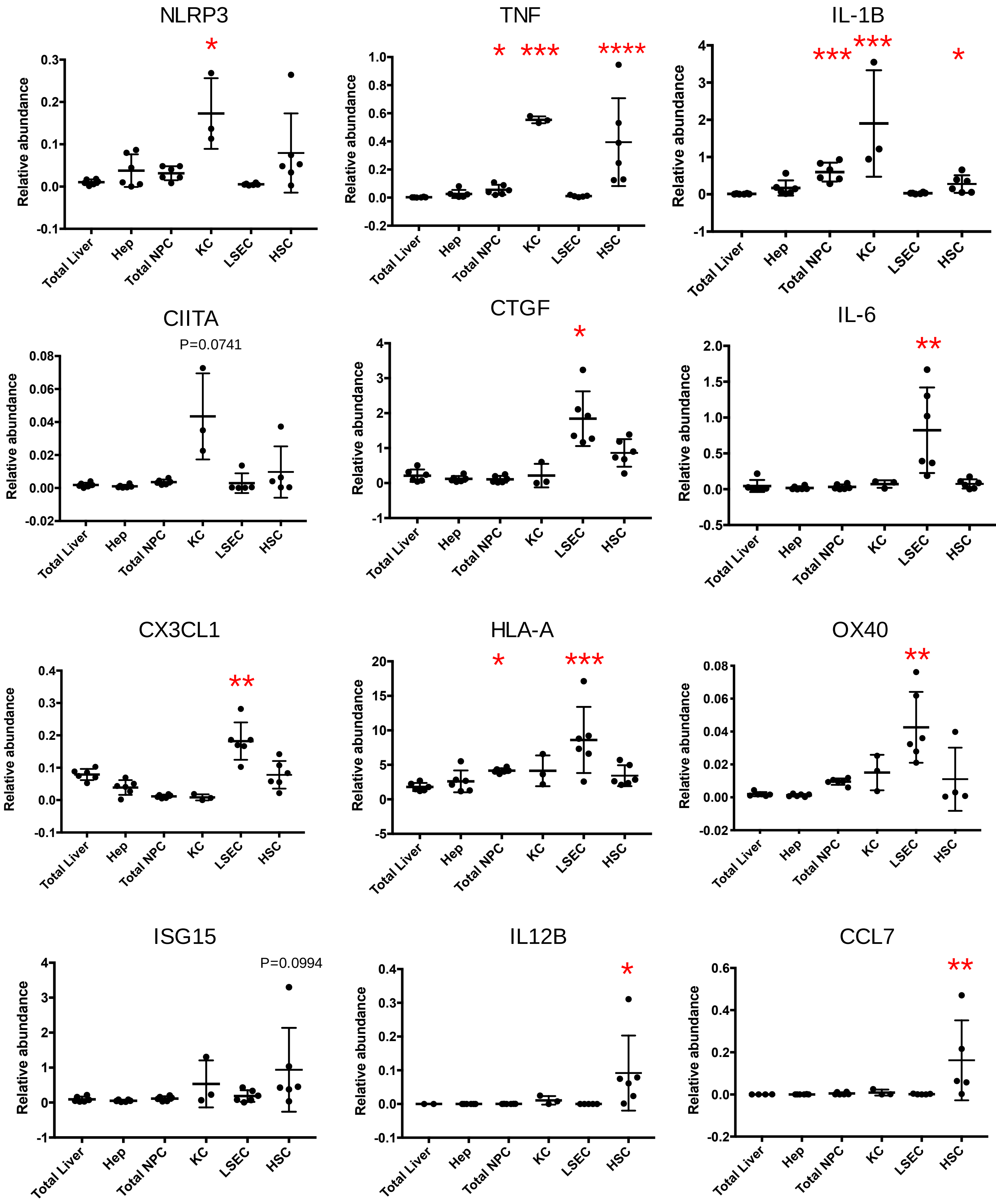
NLRP3
TNF
IL-1B
CIITA
P=0.0741
CTGF
IL-6
CX3CL1
HLA-A
OX40
ISG15
P=0.0994
IL12B
CCL7
Relative abundance
Total Liver
Hep
Total NPC
KC
LSEC
HSC

Figure S4

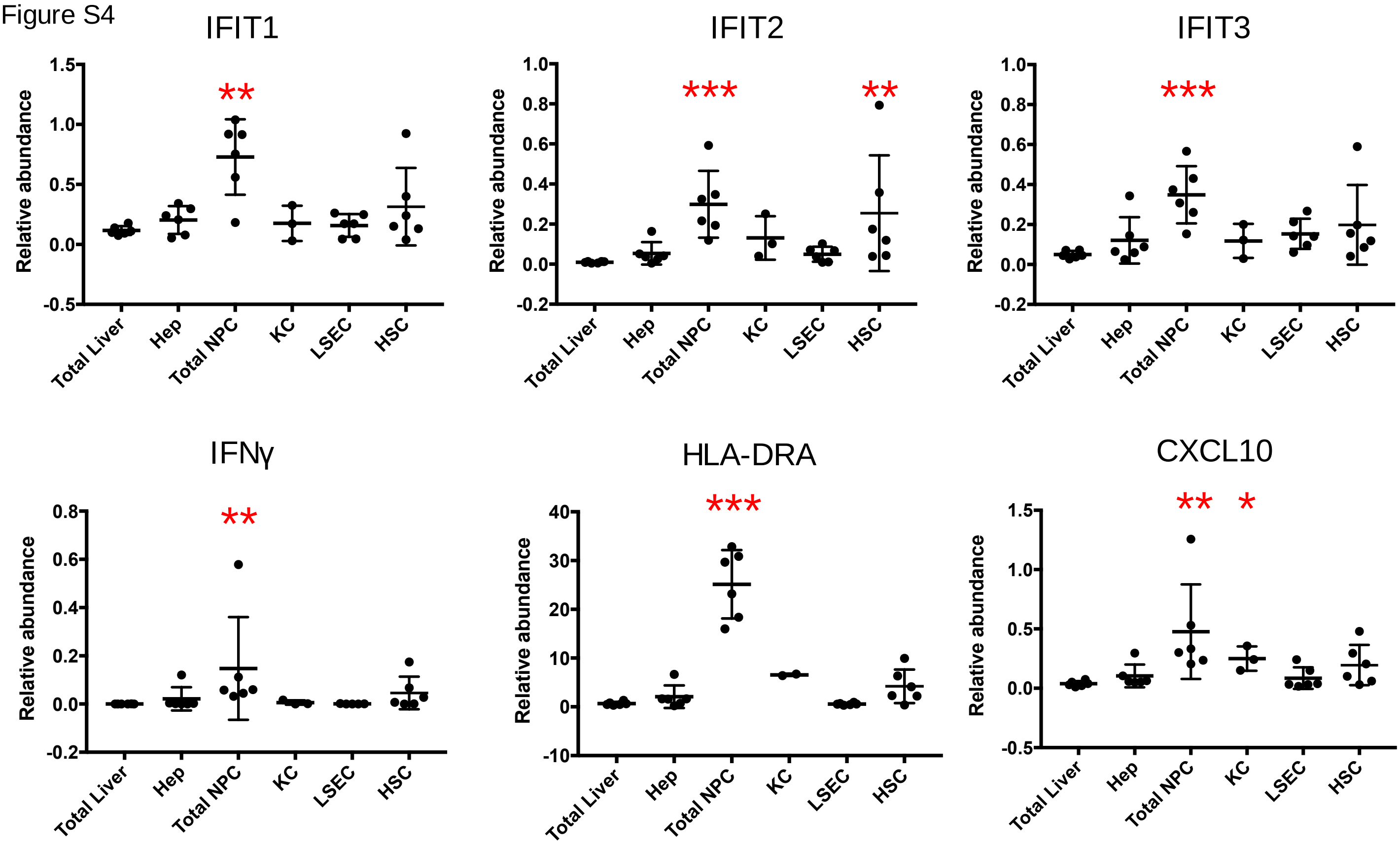

Figure S4. Gene expression detected with FACS purified liver cells. Hepatocytes (Hep), total non-parenchymal cells (NPC), Kuffer cells (KC), liver sinusoidal endothelial cells (LSEC), and hepatic stellate cells (HSC). Cell isolation was performed on wedges of resected tissue rather than cores. Non-infected liver tissue was cell sorted and analyzed for gene expression. HCV-infected or DAA-cured patients were not analyzed due to limitations in the amount of liver tissue obtained. Each dot within each cell type represents a biological replicate. The relative gene abundance was based on the delta delta Ct of the arithmetic mean of ACTB, GAPDH and HPRT1. Statistical significance was based on Kruskal-Wallis test with Dunn's post test with multiple test comparison correction, performed in Prism 9.1.0 . Genes that were enriched in a cell type compared with total livers are denoted with **red** asterisks *, P <0.05 **, P<0.01, ***, P<0.001. The mean and standard deviation are plotted.

Figure S5

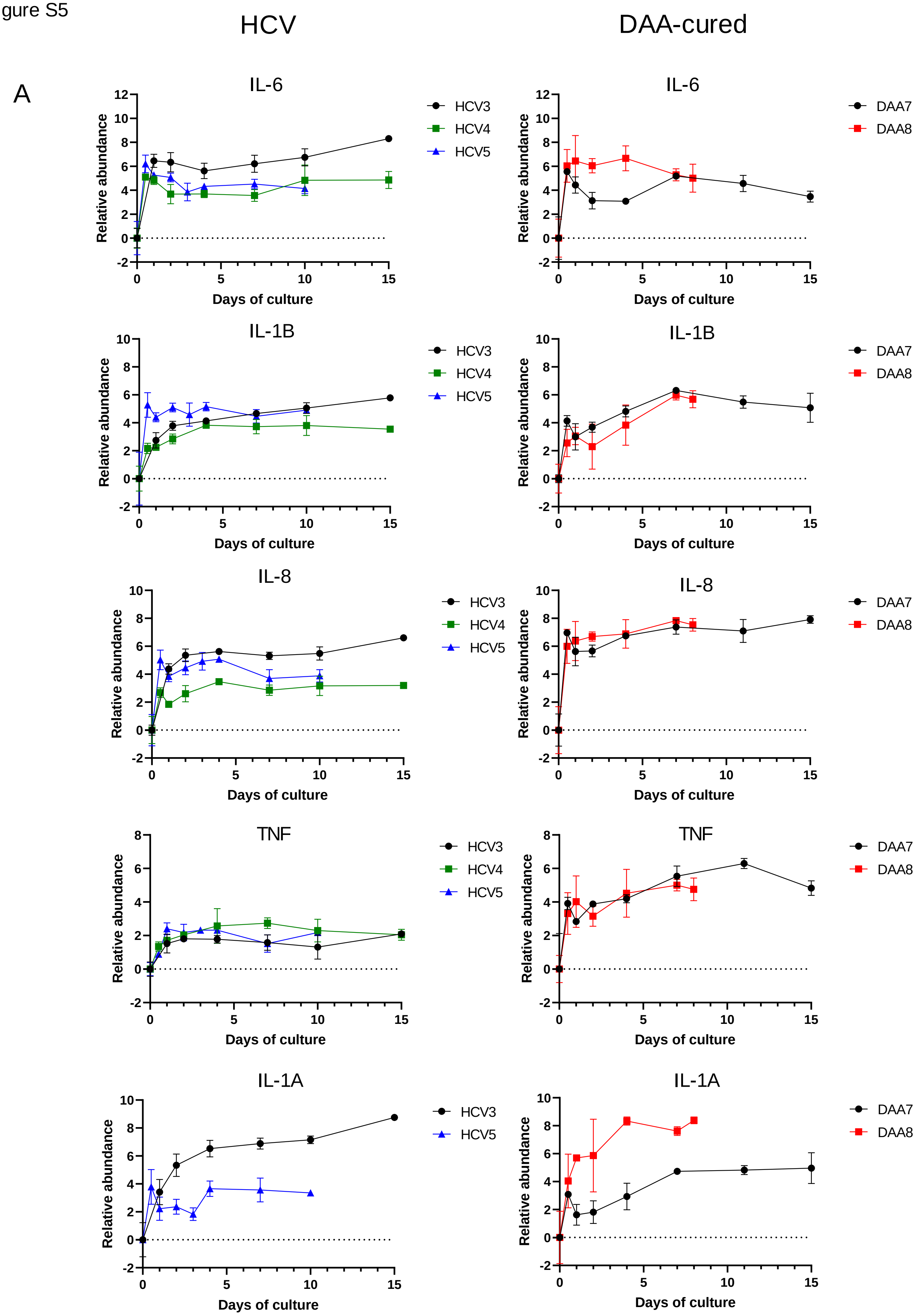
HCV
DAA-cured
A
IL-6
IL-1B
IL-8
TNF
IL-1A
Relative abundance
Days of culture
HCV3
HCV4
HCV5
DAA7
DAA8

Figure S5

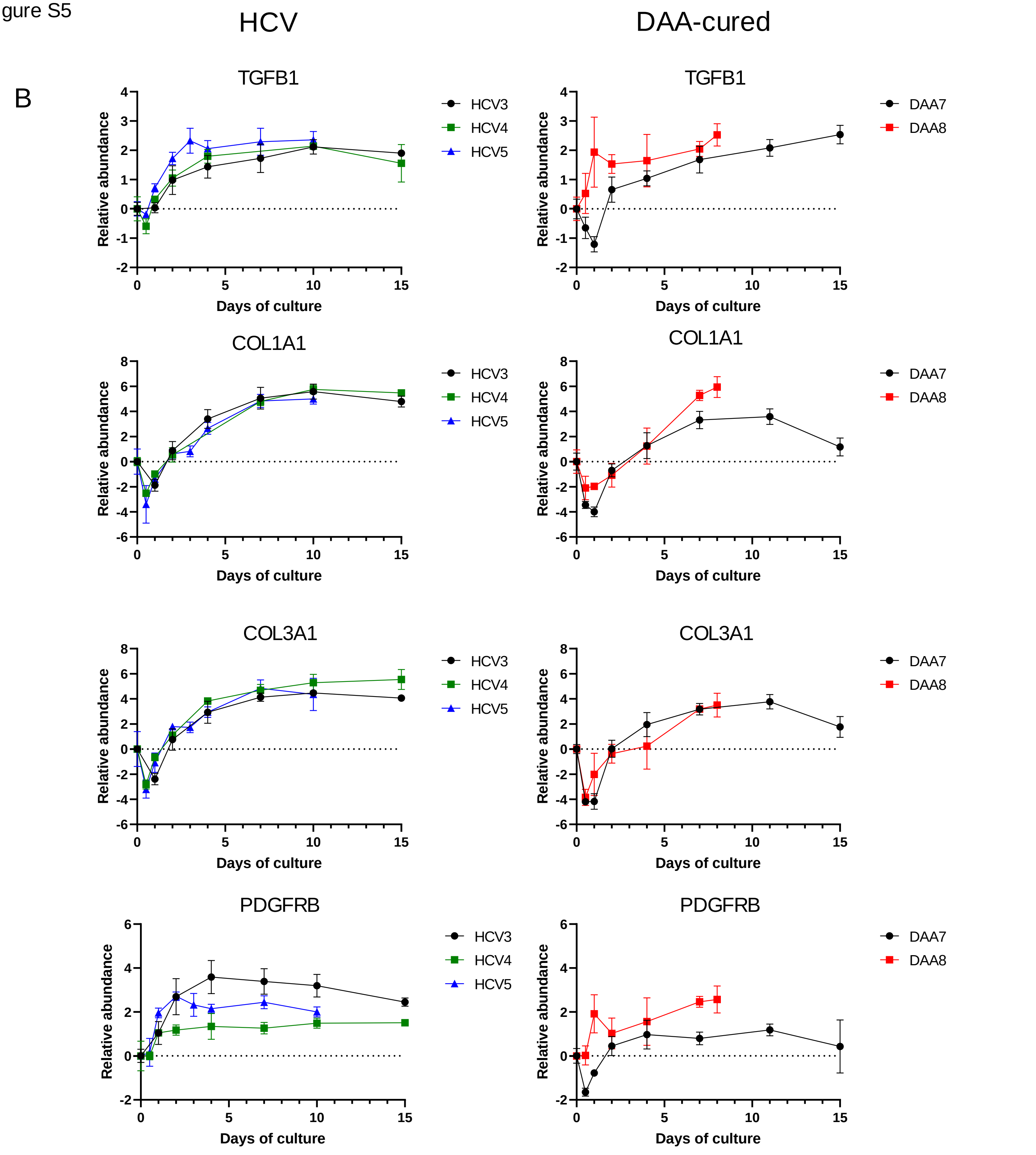

Figure S5. Inflammation and activation of repair genes post tissue slicing and culturing for HCV-infected and DAA-cured liver slices. The relative gene abundance was normalized to to the arithmetic mean of Ct values of ACTB, GAPDH and HPRT1. The relative abundance was further normalized to the day 0 measurements. (A) Inflammation genes. (B) Tissue repair genes. The mean and standard deviation for replicate slices for each time point and subject are plotted.

Figure S6

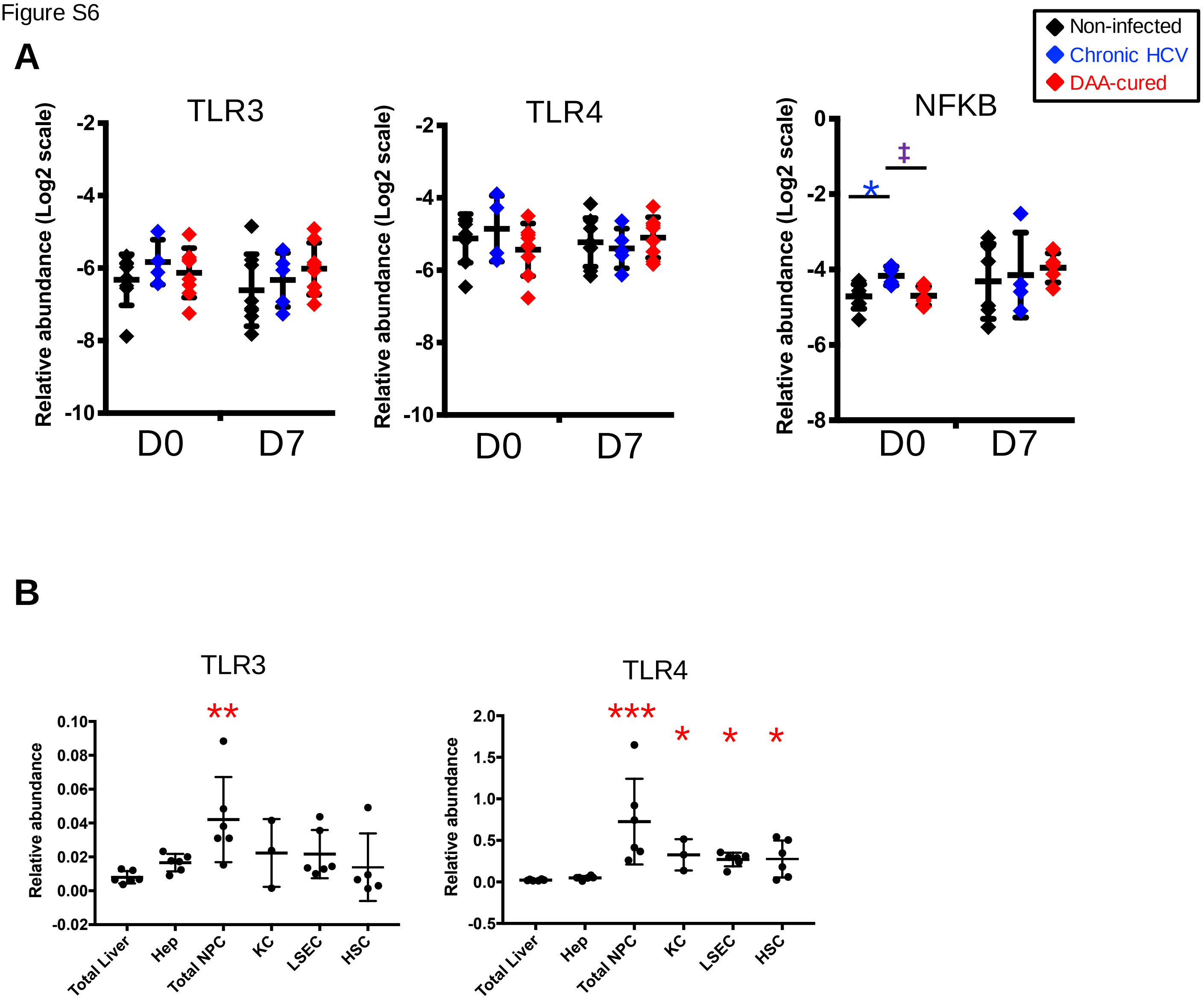

Figure S6 TLR3 and TLR4 in liver slices. (A) TLR3, TLR4 expression were not significantly altered in HCV+ and DAA-cured groups at baseline D0 and D7. NFKB expression increased in HCV+ group at Day0; seven days cultured normalized the differences. Each dot represents a patient sample. The relative gene abundance was determined with the arithmetic mean of Ct values of ACTB, GAPDH and HPRT1. Statistical significance is based on two-tailed Mann-Whitney test. *, statistical significantly different between non-infected versus chronic HCV liver slices. †, statistical significantly different between non-infected versus DAA-cured liver slices. ‡, statistical significantly different between chronic HCV versus DAA-cured liver slices. Levels of statistical significance *, P <0.05 **, P<0.01. The mean and standard deviation within each group are plotted. (B) TLR3, TLR4 expressed at NPC compartment. Each dot within each cell type represents a patient sample. The relative gene abundance was based on the delta delta Ct of the arithmetic mean of ACTB, GAPDH and HPRT1. Statistical significance is based on Kruskal-Wallis test with Dunn's post test with multiple test comparison correction. Genes that were enriched in a cell type compared with total livers are denoted with **red** asterisks *, P <0.05 **, P<0.01, ***, P<0.001. The mean and standard deviation are plotted.

Figure S7

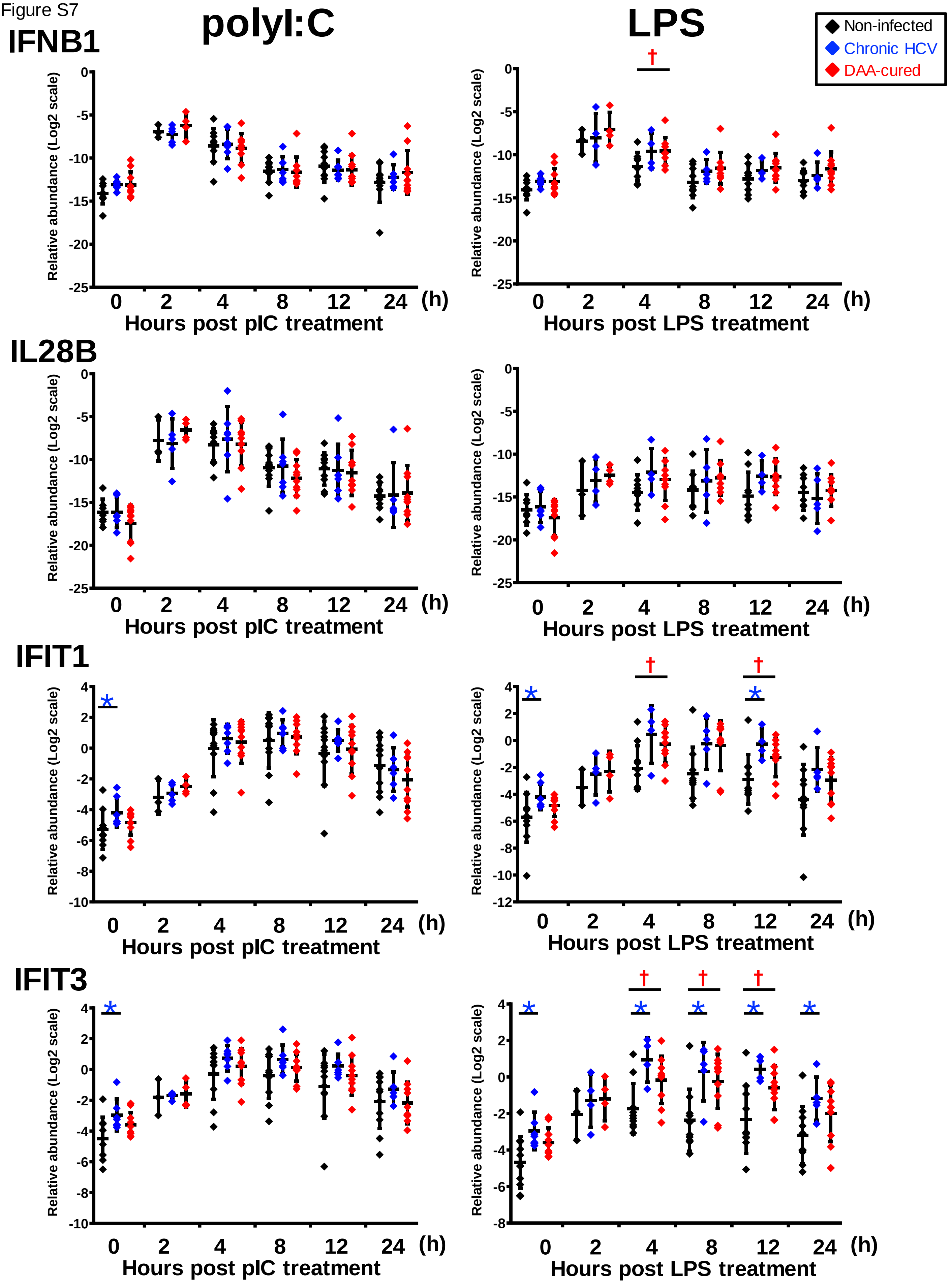
polyI:C
LPS
Non-infected
Chronic HCV
DAA-cured
IFNB1
IL28B
IFIT1
IFIT3
Relative abundance (Log2 scale)
Hours post pIC treatment
Hours post LPS treatment
0
2
4
8
12
24
(h)
†
*

Figure S7. Delta-Ct analysis showing gene responses post- poly-I:C or LPS stimulation in chronic HCV-infected and DAA-cured liver slices. The relative gene abundance was normalized to to the arithmetic mean of Ct values of ACTB, GAPDH and HPRT1. Statistical significance was based on two-tailed Mann-Whitney test. *, statistical significantly different between non-infected versus chronic HCV liver slices. †, statistical significantly different between non-infected versus DAA-cured liver slices. ‡, statistical significantly different between chronic HCV versus DAA-cured liver slices. Levels of statistical significance *, $P < 0.05$ **, $P < 0.01$. The mean and standard deviation within each group are plotted.

Figure S8

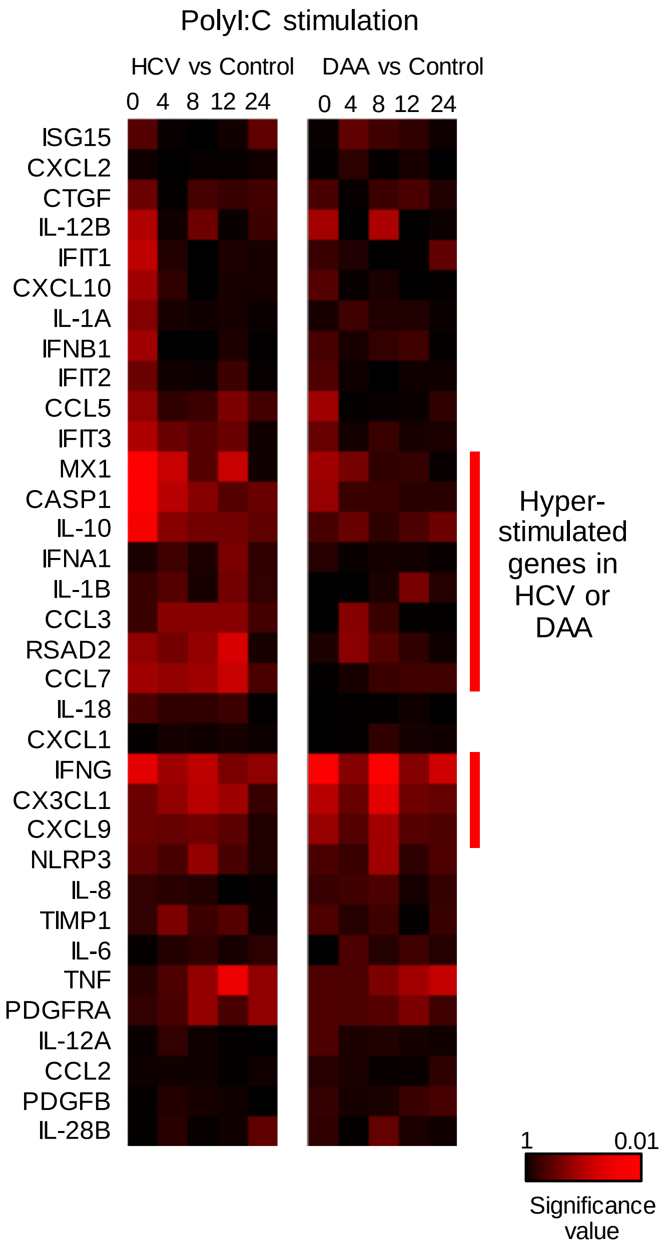

Figure S8. Altered TLR3 response in chronic HCV-infected liver slices and DAA-cured liver slices. Hierarchical clustering of immune responsive genes with 2-fold or greater induction during polyI:C stimulation. The statistical significance P value with ΔCt method is shown. Gene abundance of individual liver slices was normalized to the arithmetic mean of Ct values of ACTB, GAPDH and HPRT. Two-tailed Mann-Whitney test was used. Greater significance P value is colored with darker red. Gene clusters showing abnormality in chronic HCV-infected liver or in DAA-cured liver are highlighted.

Figure S9

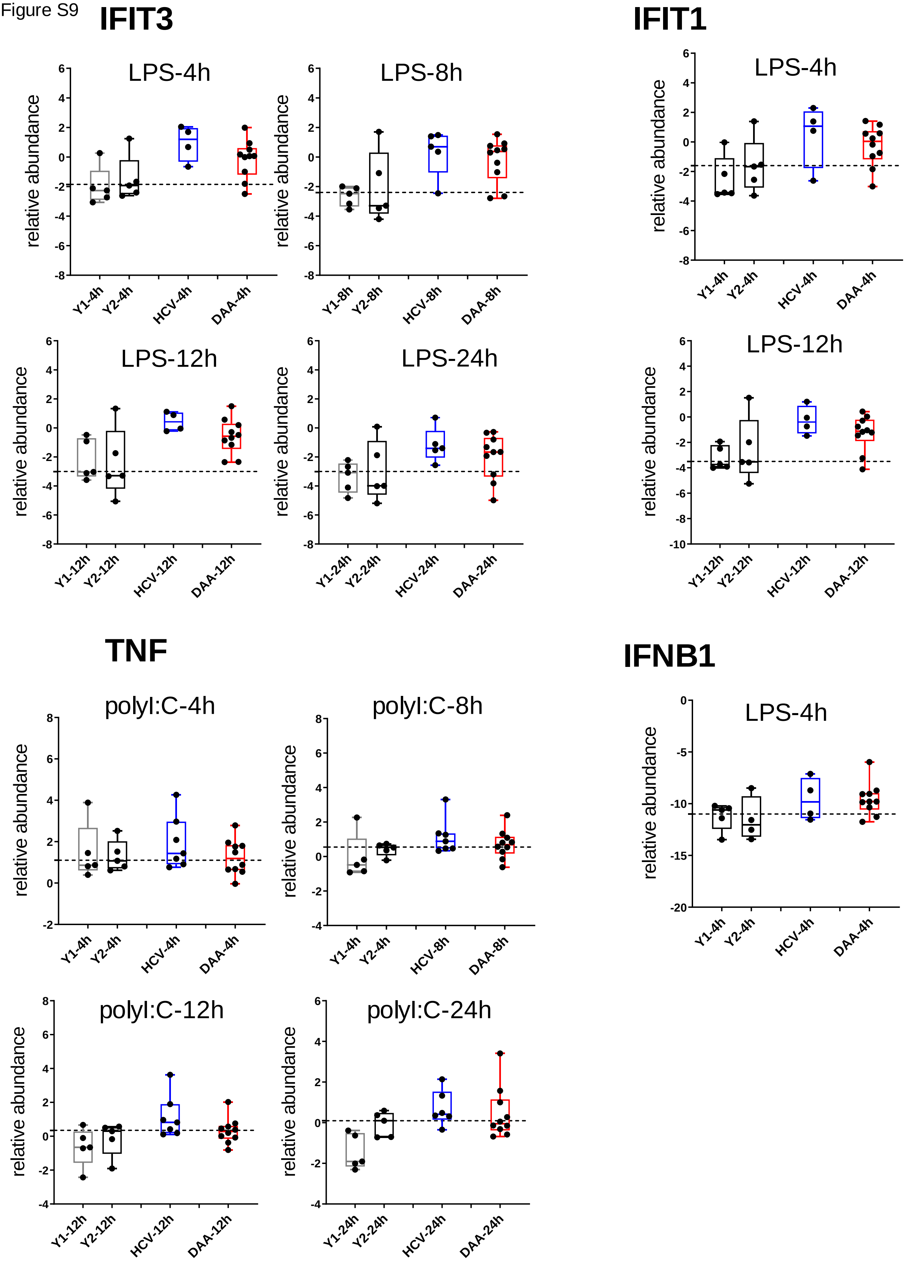

IFIT3
LPS-4h
LPS-8h
LPS-12h
LPS-24h
IFIT1
LPS-4h
LPS-12h
TNF
polyI:C-4h
polyI:C-8h
polyI:C-12h
polyI:C-24h
IFNB1
LPS-4h
relative abundance
Y1-4h
Y2-4h
HCV-4h
DAA-4h
Y1-8h
Y2-8h
HCV-8h
DAA-8h
Y1-12h
Y2-12h
HCV-12h
DAA-12h
Y1-24h
Y2-24h
HCV-24h
DAA-24h

Figure S9

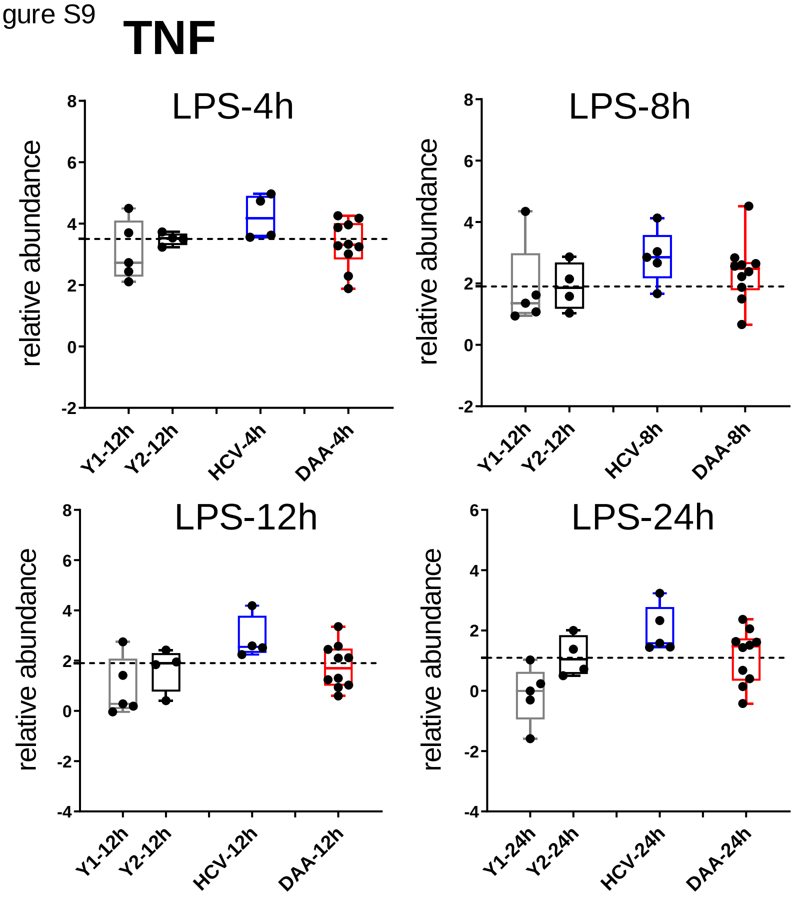

Figure S9. Comparison of gene expression of archived data with other data. Box and whiskers plots showing the median, 25%, 75% percentile range, min and max values for each group. The log2 scale of relative abundance is plotted. Y1 sample, non-infected control group (#1,3,4,5,6), from the archived data. Y2 sample, non-infected control group of the newer subjects. The dashed line is plotted with the higher of the median values within Y1 and Y2 groups for gene and time point, and is used as the reference line against the HCV+ (blue) and DAA-cured (red) corresponding data points. We did not detect statistically significant differences (defined as $P < 0.05$) between Y1 archived data and Y2 new control data, for the genes IFIT3, IFIT1, IFNB1, TNF that were differentially expressed in the HCV+ and DAA groups. The statistical test used was a two-tailed Mann-Whitney.
