# Supplementary material for: Response of Human Liver Tissue to Innate Immune Stimuli
Source: Front Immunol. 2022 Mar 9;13:811551. doi: 10.3389/fimmu.2022.811551 (PMC8959492; doi:10.3389/fimmu.2022.811551)
Supplement: Supplementary file 2 [file Table_1.pdf]

Table S1 Patient information

Chronic HCV-infected patients

Patient #	Patient ID	Age	Sex	Diagnosis	HCV genotype	HCV RNA quantitation (IU/50 ng total RNA)[1]		Prior HCV treatment
						D0	D7	
1	Chronic HCV_1	65	M	HCC	HCV G1a	3371.3	2611.5	Not treated
2	Chronic HCV_2	54	M	ICC	HCV G1a	8.2	1.8	Not treated
3	Chronic HCV_3	71	M	HCC	HCV G2	1.2	76.7	Not treated
4	Chronic HCV_4	67	M	HCC	HCV G1a	3562.7	1762.0	Not treated
5	Chronic HCV_5	66	M	HCC	HCV G1b	638.8	135.2	Not treated
6	Chronic HCV_6	71	M	HCC	HCV G1a	3279.5	405.0	Not treated
7	Chronic HCV_7	68	F	HCC	HCV G1b	9760.1	14365.8	Not treated
8	Chronic HCV_8	41	F	ICC	HCV G6	6769.8	N/A[2]	Not treated

[1]The limit of detection in the assay was 1.2 IU/50 ng liver total RNA.

[2]HCV_8 only studied the day 0 time point.

Table S1 (continued)

HCV-DAA-cured patients

Patient #	Patient ID	Age	Sex	Diagnosis	HCV genotype[1]	HCV RNA quantitation (IU/50ng total RNA)	DAA[3] treatment duration	Time from completion of DAA treatment to HCC surgery with sample collection[4]
						D0		
1	HCV-DAA_1	77	F	HCC	HCV G1b	non-detect	12 weeks	9 months
2	HCV-DAA_2	65	F	HCC	N/A	non-detect	N/A	N/A
3	HCV-DAA_3	58	M	HCC	HCV G1a	non-detect	12 weeks	10 months
4	HCV-DAA_4	62	M	HCC	HCV G1a	non-detect	24 weeks	12 months
5	HCV-DAA_5	59	F	HCC	HCV G1b	non-detect	12 weeks	7 months
6	HCV-DAA_6	77	M	HCC/ICC	N/A	non-detect	8 weeks	less than 1 month
7	HCV-DAA_7	62	F	ICC	HCV G1a or G1b	non-detect	24 weeks	9 months
8	HCV-DAA_8	62	F	HCC	HCV G1a	non-detect	12 weeks	less than 1 month
9	HCV-DAA_9	60	M	HCC	HCV G1a	non-detect	12 weeks	20 months
10	HCV-DAA_10	69	M	HCC	N/A	0.3, Below LoD[2]	12 weeks	9 months

[1]Genotype was obtained with clinical information prior to DAA treatment. N/A referred to HCV of unknown genotypes. Such patients were documented with HCV infection, however without genotype information.

[2]The 0.3 IU/50 ng liver total RNA was below the limit of detection (LoD), which was 1.2 IU/50 ng liver total RNA. Thus, this patient is HCV negative.

[3]The DAA group was treated with ledipasvir/sofosbuvir (Harvoni), which is IFN-free therapy.

[4]This indicates the time from completion of DAA treatment to HCC surgery when the liver specimens were collected and analyzed.

Table S1 (continued)

Non-infected patients

Patient #	Patient ID	Age	Sex	Diagnosis	HCV RNA quantitation (IU/50ng total RNA)
					**D0**
1	Non-infected_1[a]	67	F	Mullerian tumor	non-detect
2	Non-infected_2	75	M	ICC	non-detect
3	Non-infected_3[a]	36	M	Cholangiocarcinoma	non-detect
4	Non-infected_4[a]	62	M	Colorectal cancer	non-detect
5	Non-infected_5[a]	35	F	Hepatocellular carcinoma	non-detect
6	Non-infected_6[a]	41	F	ICC	non-detect
7	Non-infected_7	65	F	Neuroadenoma metastasis	non-detect
8	Non-infected_8	52	F	CRC	non-detect
9	Non-infected_9	33	F	Angiomyolipoma	non-detect
10	Non-infected 10	46	F	Hepatic adenoma	0.3, Below LoD[1]
11	Non-infected 11	57	F	CRC	non-detect

[a]The specimens of these patients were published previously in (Wu et al. J Immunol Methods 2018). The gene expression data are incorporated into the current manuscript. Because new non-infected controls are added, we are now in a position to draw stronger conclusions from this extended dataset, far beyond those drawn from the proof-of-concept experiments we published previously (Wu et al, 2018). This justifies the re-analysis of the same samples as part of larger groups.

[1]The 0.3 IU/50 ng liver total RNA was below the limit of detection (LoD), which was 1.2 IU/50 ng liver total RNA. Thus, this patient is HCV negative.
